# EZH2 K63-polyubiquitination affecting migration in extranodal natural killer/T-cell lymphoma

**DOI:** 10.1186/s13148-023-01606-6

**Published:** 2023-11-29

**Authors:** Boheng Li, Qidi Zhou, Qin Wan, Xuan Qiao, Shangying Chen, Jianbiao Zhou, Zhijun Wuxiao, Lei Luo, Siok-Bian Ng, Jieping Li, Wee-Joo Chng

**Affiliations:** 1 College of Pharmaceutical Sciences, Southwest University, Chongqing, China; 2 Bioinformatics Core, Yong Loo Lin School of Medicine, National University of Singapore, Singapore, Singapore; 3 Cancer Science Institute of Singapore, National University of Singapore, Singapore, Singapore; 4 Department of Hematology, Lymphoma and Myeloma Center, The First Affiliated Hospital of Hainan Medical University, Haikou, China; 5 Department of Pathology, Yong Loo Lin School of Medicine, National University of Singapore, Singapore, Singapore; 6 Department of Hematology Oncology, Chongqing University Cancer Hospital, Chongqing, China; 7 Department of Medicine, Yong Loo Lin School of Medicine, National University of Singapore, Singapore, Singapore; 8 Department of Hematology-Oncology, National University Cancer Institute of Singapore, National University Health System, Singapore, Singapore

**Keywords:** Extranodal natural killer/T-cell lymphoma, Migration, EZH2, TRIP12, K63-polyubiquitination

## Abstract

**Background:**

Overexpressed EZH2 is oncogenically involved in the pathogenesis of different cancerous contexts including extranodal natural killer/T cell lymphoma (ENKTL). However, the underlying mechanisms of EZH2 upregulation have not been fully clarified and it is still difficult to target EZH2 in ENKTL.

**Results:**

Current study identifies an E3 ligase TRIP12 that triggers K63-linked polyubiquitination of EZH2 in ENKTL and unexpectedly, stabilizes EZH2. As determined by gene expression profiling (GEP), TRIP12 and EZH2 levels correlate with each other in ENKTL patient samples. Aided by quantitative mass spectrometry (MS) and follow-up analysis, we identify K634 as the ubiquitination site of EZH2. Further study confirms that TRIP12-mediated EZH2 K634 ubiquitination enhances the interaction between EZH2 and SUZ12 or CDK1 and increases the level of EZH2 T487 phosphorylation. This study further demonstrates the TRIP12-EZH2 signaling might be regulated by cytoplasmic HSP60. Importantly, the TRIP12-EZH2 axis mediates ENKTL cell migration via accelerating epithelial-mesenchymal transition (EMT). Moreover, our study finds out dexamethasone treatment manipulates TRIP12-EZH2 signaling and may represent a novel therapeutic strategy against ENKTL metastasis.

**Conclusions:**

Altogether, TRIP12 induces K63-linked site-specific polyubiquitination of EZH2 for stabilization, which promotes ENKTL cell migration and could be targeted by dexamethasone treatment.

**Supplementary Information:**

The online version contains supplementary material available at 10.1186/s13148-023-01606-6.

## Background

EZH2 is the enzymatic subunit of the polycomb repressive complex 2 (PRC2), and catalyzes histone H3 tri-methylation at K27 site, therefore turning off gene expression. As PRC2 is a holoenzyme, EZH2 has to gather with other subunits like SUZ12 and EED to realize its enzymatic activity. Overexpression of EZH2 has been identified in a variety of neoplasms, which often accelerates tumor progression and associates with poor clinical outcomes. Of note, EZH2 overexpression may directly modulate the process of epithelial-mesenchymal transition (EMT) and therefore affect tumor cell migration and invasion [1, 2].

However, the underlying mechanisms of EZH2 overexpression are multi-faceted and remain poorly understood, among which the deregulation of the ubiquitin–proteasome system (UPS) is indispensable and context-dependent. Aberrant expressions of E3 ligases and deubiquitinases both contribute to UPS malfunction and abnormal EZH2 expression. K48- and K63-linked ubiquitination are commonly known as two most prevalent topologies of polyubiquitination. E3s including FBW7 [3], c-Cbl [4], β-TrCP [5], Smurf2 [6] and CHIP [7] were all reported to catalyze proteolytic and mostly K48-linked ubiquitination of EZH2 in different cellular contexts, and we showed previously that the deubiquitinase USP36 removed the ubiquitin tag to stabilize EZH2 which was a downstream event following MELK-mediated EZH2 phosphorylation in extranodal natural killer/T cell lymphoma (ENKTL) [8]. Importantly, K63-linked polyubiquitination of EZH2 initiating other events rather than directly sending EZH2 to proteasomal degradation was scarcely seen regardless of disease context.

In most occasions, EZH2 exhibits oncogenicity through mediating H3K27 tri-methylation followed by transcriptional repression. However, in several highly malignant and lethal cancers including ENKTL, the oncogenic role of EZH2 is frequently H3K27 tri-methylation independent. EZH2 may associate with other transcription-related factors to turn on gene expression rather than transcription suppression [9–11]. A couple of small-molecule inhibitors of EZH2 were successively commercialized by pharmaceutical manufacturers and these inhibitors specifically compete with catalytic SET domain to inhibit enzymatic activity of EZH2 but leave pan-EZH2 unchanged [12, 13]. Therefore, the abovementioned inhibitors cannot block EZH2-mediated tumorigenicity in ENKTL. In recent years, peptide inhibitor SAH-EZH2 [14] and EZH2 degrader MS1943 [15] claiming to be able to remove total cellular EZH2 were reported. Unfortunately, both SAH-EZH2 [9] and MS1943 (unpublished) were unable to degrade EZH2 level in ENKTL cells. Therefore, it is urgent to investigate underlying mechanisms mediating EZH2-initiated oncogenesis for the purpose of developing novel therapeutics to effectively harness EZH2 in ENKTL.

TRIP12 is commonly known as an oncogenic E3 ligase capable of degrading several tumor suppressors and thus has a role in mediating cell cycle progression and chemo-resistance [16, 17]. HSP60 is usually considered as a chaperone protein that conventionally locates in the mitochondria to maintain protein homeostasis [18]. In this study, we demonstrate that in ENKTL, TRIP12 catalyzes K63-linked ubiquitination of EZH2 for EZH2 stabilization, which is regulated by upstream cytoplasmic HSP60, accelerating ENKTL cell migration via inducing EMT. It is reported that IDO is a rate-limiting enzyme of tryptophan metabolism [19]. Dexamethasone, an agonist of IDO, thus promotes metabolism of tryptophan to destabilize downstream TRIP12 and EZH2 and effectively obstructs ENKTL cell migration. These findings delineate a novel mechanism elucidating EZH2 overexpression in ENKTL, hint that TRIP12 might be a therapeutic target for hematologists to combat against ENKTL cell migration and uncover that conventional steroid therapy might prevent ENKTL metastasis via specifically targeting TRIP12-EZH2 signaling.

## Results

### TRIP12 interacts with and stabilizes overexpressed EZH2

**Fig. 1:**
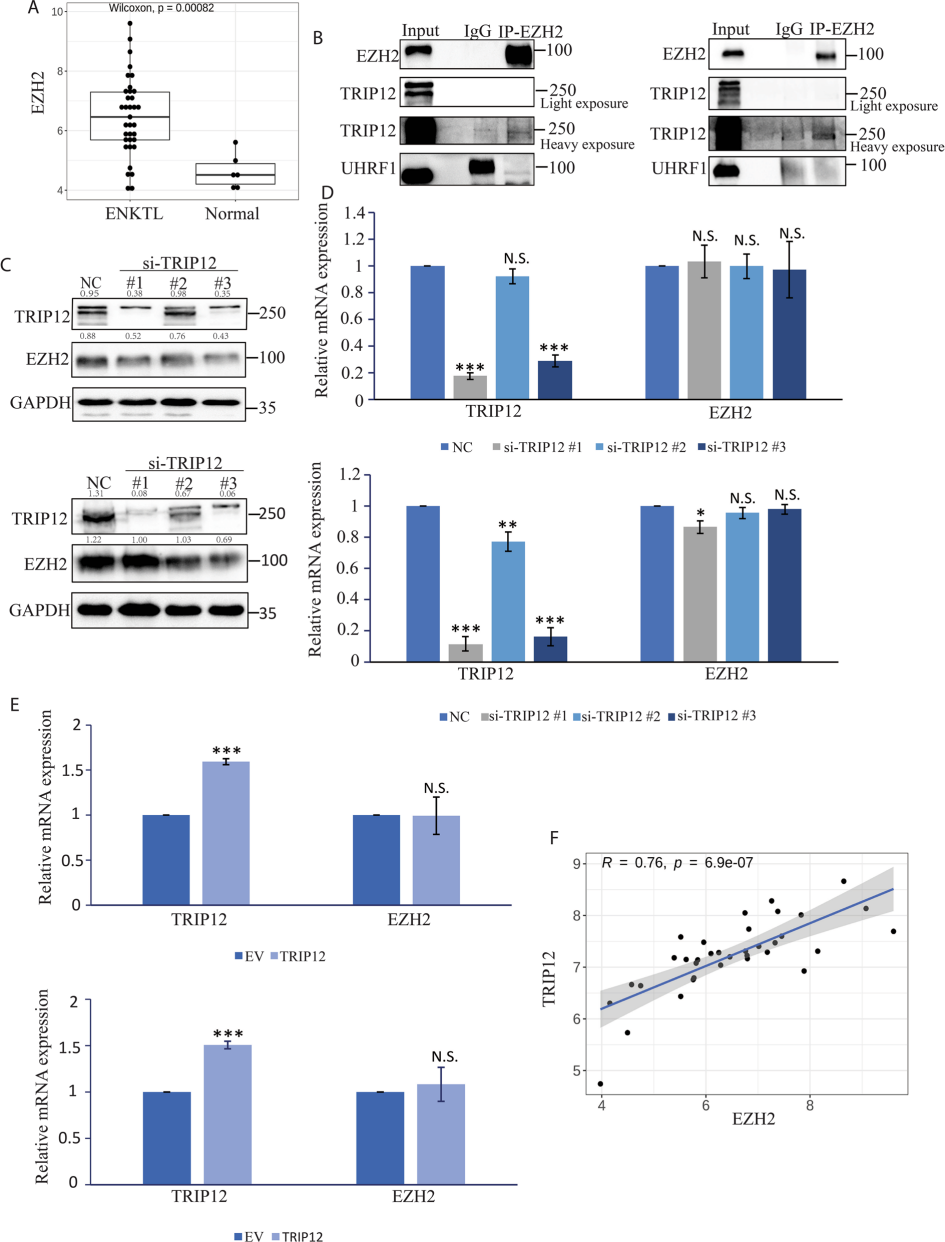
E3 ligase TRIP12 correlates with EZH2 expression in extranodal natural killer/T-cell lymphoma (ENKTL). **A** Gene expression profiling (GEP) data showing EZH2 expression in ENKTL: 35 patient samples and 6 normal control tissues. **B** Co-IP showing interaction between EZH2 and TRIP12 or UHRF1 in YT (left) and NK92 cells (right). **C** EZH2 protein level change upon TRIP12 knockdown using siRNA in YT (upper) and NKYS (lower) cells. N = 3 individual experiments and representative images are shown. ENKTL cells were harvested for immunoblots 48 h after knockdown with Neon transfection system. **D** EZH2 mRNA level change upon TRIP12 knockdown using siRNA in YT (upper) and NKYS (lower) cells. ENKTL cells were harvested for RNA extraction 24 h after knock down. **E** EZH2 mRNA level change upon Flag-TRIP12 or empty vector transfection in YT (upper) and NKYS (lower) cells. The cells were harvested for RNA extraction 16 h after overexpression. Results are mean ± SD. N = 3; N.S. not significant; *p < 0.05; **p < 0.01; ***p < 0.001. **F** linear correlation between TRIP12 and EZH2 expression obtained from GEP data of 35 ENKTL patient samples (R = 0.76, *p* = 6.9e−07)

As indicated by GEP analysis, EZH2 was significantly overexpressed in ENKTL patient samples compared with normal counterparts (*p* = 0.00082, Fig. 1A). In order to investigate the underlying mechanisms leading to EZH2 overexpression, we examined EZH2 interactome data in ENKTL (unpublished) and found that two E3 ligases TRIP12 and UHRF1 were considered as possible interacting proteins of EZH2, which might be potentially responsible for EZH2 turnover. Only the interaction between EZH2 and TRIP12 was confirmed through co-IP in ENKTL cell lines (Fig. 1B). TRIP12 level was then genetically manipulated to check whether there was a mediation on EZH2. Unexpectedly, siRNA-mediated TRIP12 knockdown resulted in a decrease rather than an increase of EZH2 at the protein level (Fig. 1C). And both TRIP12 knockdown and overexpression did not hinder or promote the mRNA level of EZH2 (Fig. 1D, E). TRIP12 and EZH2 expressions were significantly correlated with each other in ENKTL patient samples (R = 0.76, *p* = 6.9e−07, Fig. 1F). These results demonstrate the internal association between TRIP12 and EZH2 in ENKTL and TRIP12 may modulate EZH2 turnover unlike a conventional E3 ligase.

### TRIP12 promotes ENKTL cell survival

**Fig. 2:**
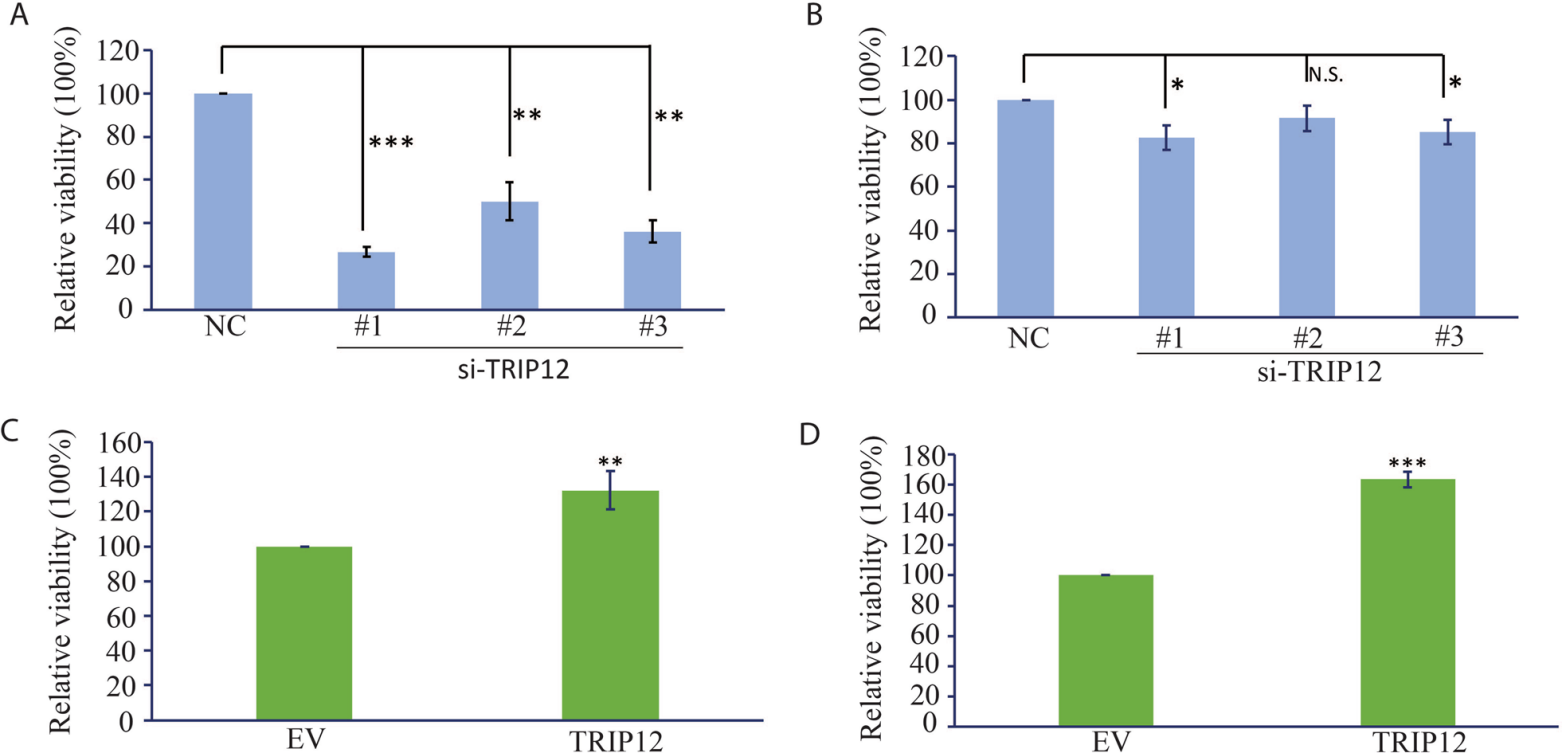
Role of TRIP12 in mediating ENKTL cell survival. **A**, **B** Relative cell viability upon TRIP12 knockdown using siRNA in YT (**A**) and NKYS (**B**). The cells were subjected to Cell Counting Kit-8 assay 48 h after knockdown. **C**, **D** Relative cell viability with TRIP12 or empty vector transfection in YT (**C**) and NKYS (**D**). The cells were subjected to Cell Counting Kit-8 assay 16 h after transfection. Results are mean ± SD. N = 3; N.S. not significant; *p < 0.05; **p < 0.01; ***p < 0.001

Since TRIP12 was found to stabilize EZH2, and EZH2, as we reported previously, conferred a survival advantage to ENKTL cells [20], we hypothesized that TRIP12 might also positively regulate ENKTL cell survival. As expected, TRIP12 knockdown decreased cell survival (Fig. 2A, B) and TRIP12 overexpression enhanced their survival (Fig. 2C, D).

### TRIP12 modulates EZH2-based transcriptional regulation

**Fig. 3:**
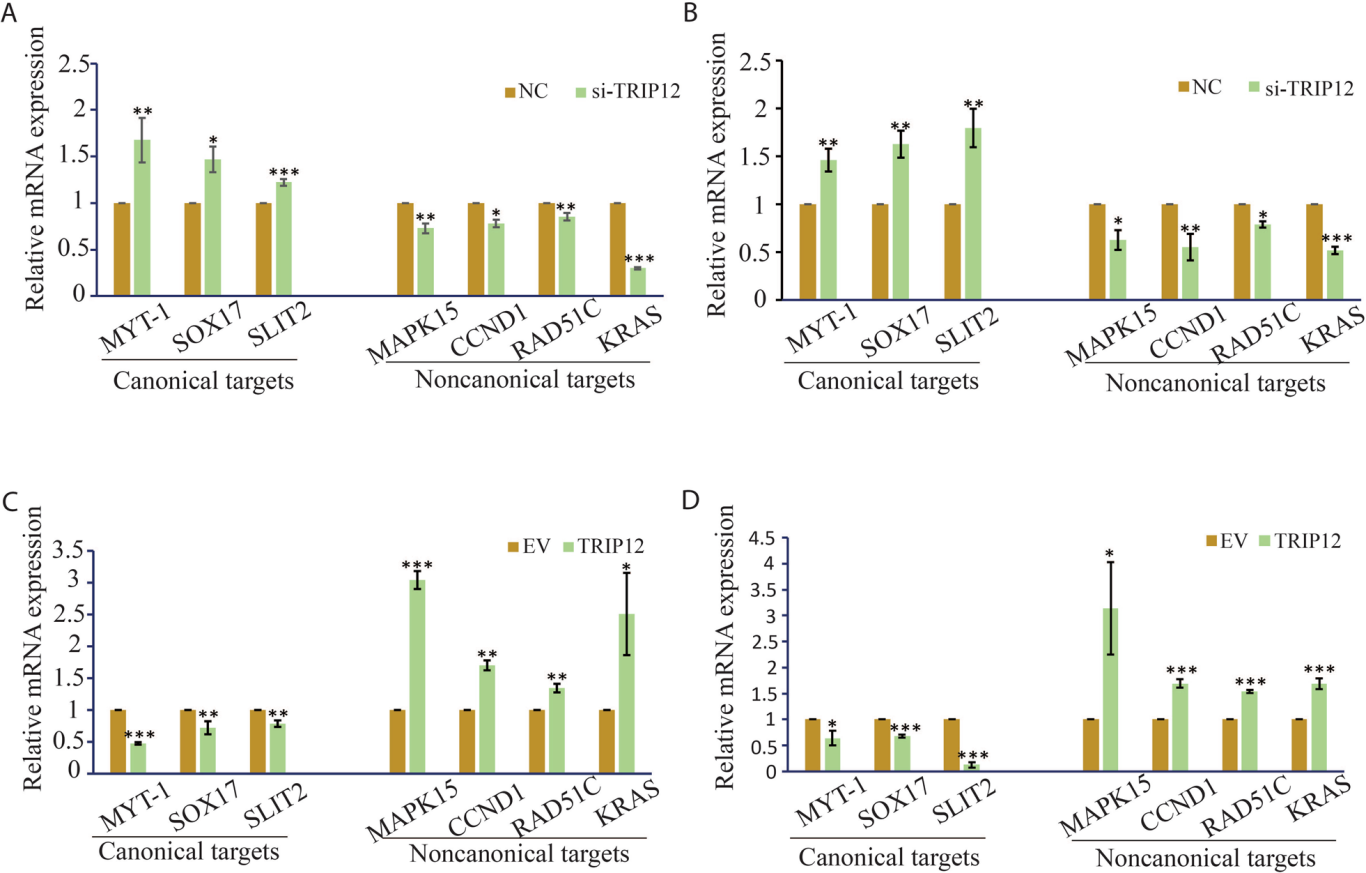
TRIP12 modulates EZH2 target gene expression. **A**, **B** Relative mRNA expression of canonically-repressed and noncanonically-activated target genes of EZH2 upon siRNA-mediated knockdown of TRIP12 in YT (**A**) and NKYS (**B**). **C**, **D** Relative mRNA expression of canonically-repressed and noncanonically-activated target genes of EZH2 upon TRIP12 or empty vector transfection in YT (C) and NKYS (**D**). Results are mean ± SD. N = 3; N.S. not significant; *p < 0.05; **p < 0.01; ***p < 0.001

As we demonstrated before, in the cellular context of ENKTL, EZH2 canonically repressed the expression of a set of genes via H3K27 tri-methylation, including MYT-1, SOX17 and SLIT2, and noncanonically trans-activated another gene set (MAPK15, CCND1, RAD51C and KRAS) in mediating oncogenesis [9]. Therefore, in this study, we examined whether TRIP12 expression also affected these gene sets. As illustrated, all canonically-repressed genes showed increased expression and those noncanonically-activated genes showed expression decrease upon TRIP12 knockdown (Fig. 3A, B). An opposite pattern of expression alterations was observed with TRIP12 overexpression (Fig. 3C, D). These results reveal TRIP12 mediates EZH2 transcriptional behaviors possibly via suppressing EZH2 turnover in ENKTL.

### TRIP12 triggers K63-linked ubiquitination of EZH2

**Fig. 4:**
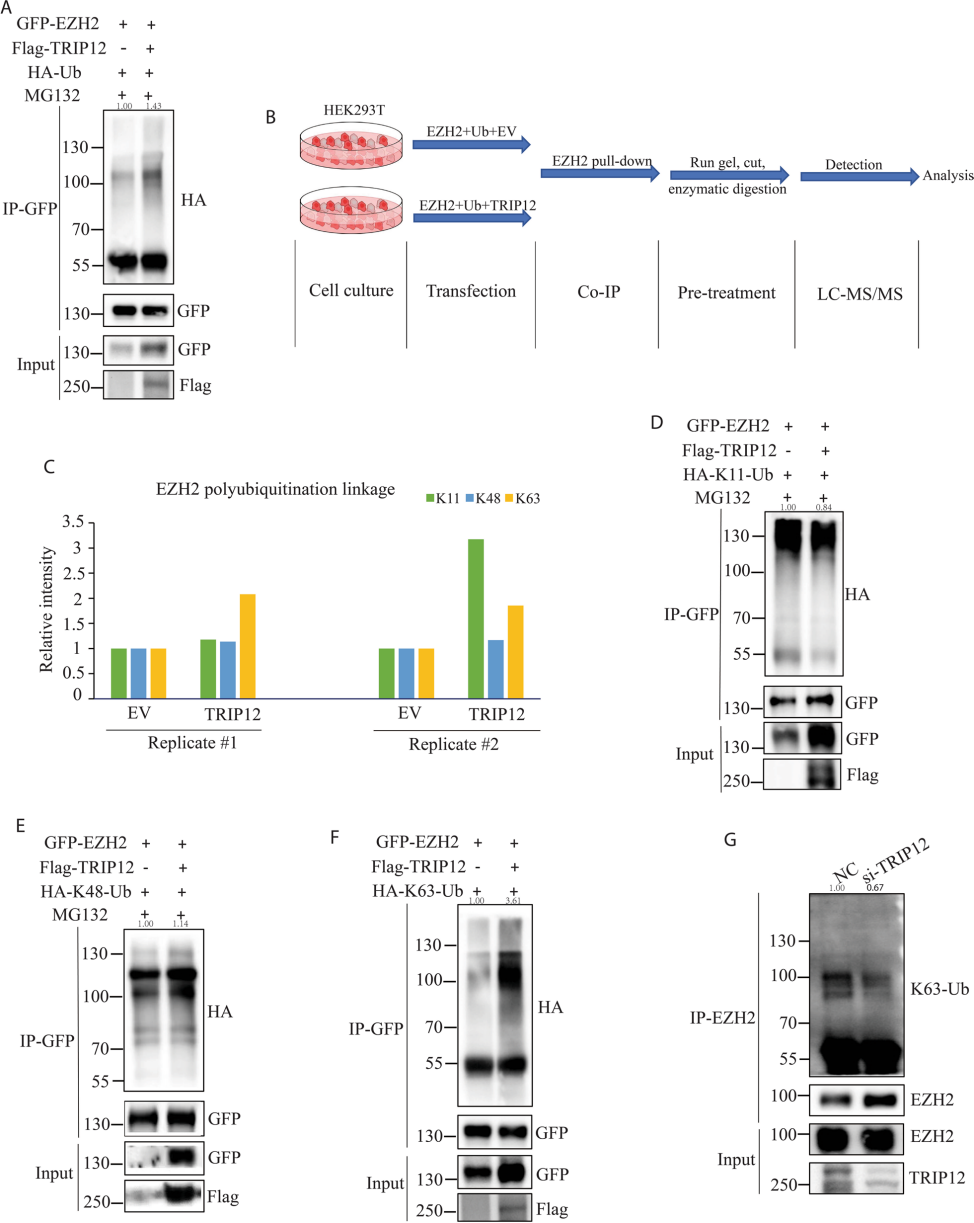
TRIP12 initiates K63-linked polyubiquitination of EZH2. **A** Change of EZH2 ubiquitination level with TRIP12 overexpression in HEK293T cells. MG132 was given at 5 µM for 6 h of treatment 48 h after transfection. **B** Schematic protocols showing steps of LC–MS/MS analysis. N = 2 individual experiments. **C** Bar chart showing change of K11-, K48- and K63-linked polyubiquitination with TRIP12 overexpression in HEK293T cells which was identified by LC–MS/MS analysis. **D**, **E** Change of EZH2 K11- (**D**) or K48- (**E**) linked polyubiquitination with TRIP12 overexpression in HEK293T cells. MG132 was given at 5 µM for 6 h of treatment 48 h after transfection. **F** Change of EZH2 K63-linked polyubiquitination with TRIP12 overexpression in HEK293T cells. **G** Change of EZH2 K63-linked polyubiquitination upon siRNA-mediated TRIP12 knockdown in NKYS cells. N = 3 individual experiments for all Co-IPs and representative images are shown

Given that TRIP12 is an E3 ligase and TRIP12 affected EZH2 protein turnover, TRIP12 may mediate EZH2 ubiquitination. As shown in Fig. 4A, overexpression of TRIP12 led to a clear increase of EZH2 ubiquitination. Next, to confirm the role of TRIP12 in EZH2 ubiquitination, EZH2 was immunoprecipitated from 293T cells transfected with ubiquitin, EZH2 and either TRIP12 or empty vector for LC–MS/MS analysis (Fig. 4B). Three types of EZH2 polyubiquitination, K11, K48 and K63 linkages were identified through MS, but only K63-linked EZH2 polyubiquitination levels always exhibited increase upon TRIP12 overexpression (Fig. 4C). To further confirm EZH2 polyubiquitination linkage, we overexpressed EZH2, K11/K48/K63-ubiquitin and TRIP12/empty vector and consistently observed that TRIP12 overexpression only increased K63-linked EZH2 polyubiquitination with K11 and K48 polyubiquitination almost unchanged (Fig. 4D–F). When TRIP12 was knocked down, a decrease of EZH2 K63-linked polyubiquitination was seen (Fig. 4G). Taken together, these results corroborate TRIP12 is responsible for EZH2 K63-linked polyubiquitination.

### TRIP12 specifically ubiquitinates EZH2 at K634

**Fig. 5:**
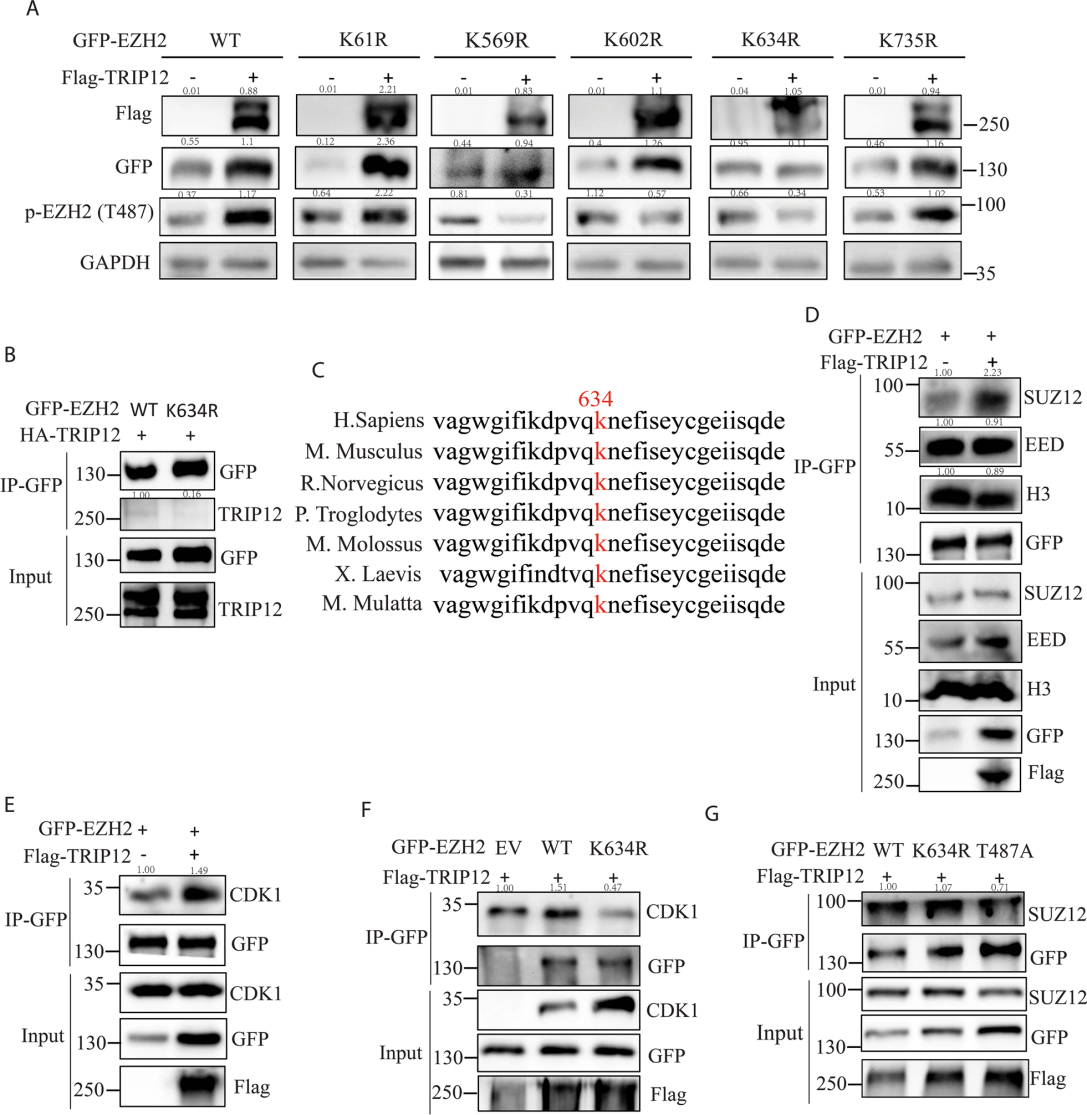
TRIP12 mediates EZH2 K634 ubiquitination and meanwhile enhances PRC2 stability and T487 phosphorylation. **A** Change of exogenously overexpressed EZH2 and endogenous p-EZH2 (T487) level with indicated transfections. **B** Co-IP showing change of interaction between TRIP12 and EZH2 mutant. **C** Schematic diagram indication: the conservation of EZH2 K634. (D) Co-IP showing change of interaction between EZH2 and PRC2 components or histone H3 with TRIP12 overexpression. **E** Co-IP showing change of interaction between EZH2 and CDK1 with or without TRIP12 overexpression. **F** Co-IP showing change of interaction between CDK1 and EZH2 mutant with TRIP12 overexpression. **G** Co-IP showing change of interaction between EZH2 or its mutants and SUZ12 with TRIP12 overexpression. All immunoblots or co-IPs were performed in HEK293T cells, and the cells were harvested for lysis 48 h after transfection. N = 3 individual experiments and representative images are shown

Then we generated ubiquitination-null mutants (EZH2 K61R, K569R, K602R, K634R and K735R) according to all the potential EZH2 ubiquitination sites suggested by the abovementioned LC–MS/MS data (Table 1). We examined whether TRIP12 overexpression could still stabilize these mutants. Compared with EZH2 wildtype (WT), among the mutants, only K634R transfection compromised the increase of exogenous EZH2 with TRIP12 overexpression (Fig. 5A). The EZH2 K634R mutant also showed decreased interaction with TRIP12 compared with EZH2 WT (Fig. 5B). As mentioned above, EZH2 is oncogenic in ENKTL [20], very possibly any EZH2 stability alteration could affect ENKTL cell survival. As anticipated, only EZH2 K634R was always able to reverse survival advantages conferred by EZH2 WT (Additional file 1: Fig. 1A and B). Furthermore, as we showed previously, CCND1 was trans-activated by EZH2 as part of EZH2-based oncogenesis in ENKTL [20], and EZH2 K634R mutant could rescue the increase in CCND1 mRNA expression bestowed by EZH2 WT (Additional file 1: Fig. 1C and D). Then we sought to investigate whether the K634 ubiquitination site was also critical for those canonically-repressed target genes of EZH2. As expected, EZH2 K634R mutant reversed the suppression of a set of canonical genes (MYT-1, SOX17 and SLIT2, Additional file 1: Fig. 1E and F). Of note, K634 was one of the conserved sites of EZH2 across species (Fig. 5C). Collectively, these findings indicate TRIP12 stabilizes EZH2 and promotes EZH2-mediated oncogenesis in ENKTL possibly by site-specific ubiquitination at K634.

**Table 1:** Identified EZH2 ubiquitination sites associated with TRIP12

	Site	Peptide	EV intensity	TRIP12 OE intensity
Replicate #1	K61	TEILNQEW**K**QR	5826300	18779000
Replicate #2			16212000	29238000
Replicate #1	K569	AQCNT**K**QCPCYLAVR	0	128070000
Replicate #2			61066000	132810000
Replicate #1	K602	NVSC**K**NCSIQR	0	51302000
Replicate #2			0	15051000
Replicate #1	K634	DPVQ**K**NEFISEYCGEIISQDEADRR	0	24421000
Replicate #2			0	60011000
Replicate #1	K735	YSQADAL**K**YVGIER	0	432330000
Replicate #2			160200000	359300000

### TRIP12-mediated EZH2 ubiquitination enhances PRC2 cohesion and T487 phosphorylation

Next, we investigated how TRIP12-mediated EZH2 K634 ubiquitination affected enzymatic activity and other post-translational modification of EZH2. With TRIP12 overexpression, EZH2 displayed increased interaction with another PRC2 subunit SUZ12 and unchanged binding with PRC2-EED and histone H3 (Fig. 5D), suggesting enhanced PRC2 cohesion and potentially enhanced enzymatic activity. CDK1 was one of the interacting proteins that demonstrated increased binding with EZH2 upon TRIP12 overexpression as suggested by the abovementioned MS data. And this was confirmed by EZH2 co-IP (Fig. 5E). When the ubiquitination site of EZH2 was mutated, the binding to CDK1 decreased (Fig. 5F). Previous studies have well characterized CDK1 as a kinase to catalyze EZH2 T487 phosphorylation [21, 22]. Here, we observed increased T487 phosphorylation with TRIP12 overexpression co-transfected with EZH2 WT, and this increase was totally rescued with EZH2 K634R co-transfection, indicating K634 was necessary for CDK1-mediated phosphorylation (Fig. 5A). To further characterize the T487 phosphorylation site, we generated EZH2 phosphorylation-null T487A mutant. Importantly, T487A displayed attenuated interaction with SUZ12 (Fig. 5G) as expected. These findings demonstrate that TRIP12-mediated EZH2 polyubiquitination at K634 increases its interaction with PRC2-SUZ12 and promotes CDK1-catalyzed T487 phosphorylation.

### Heat shock protein (HSP) modulates TRIP12 and EZH2 signaling

**Fig. 6:**
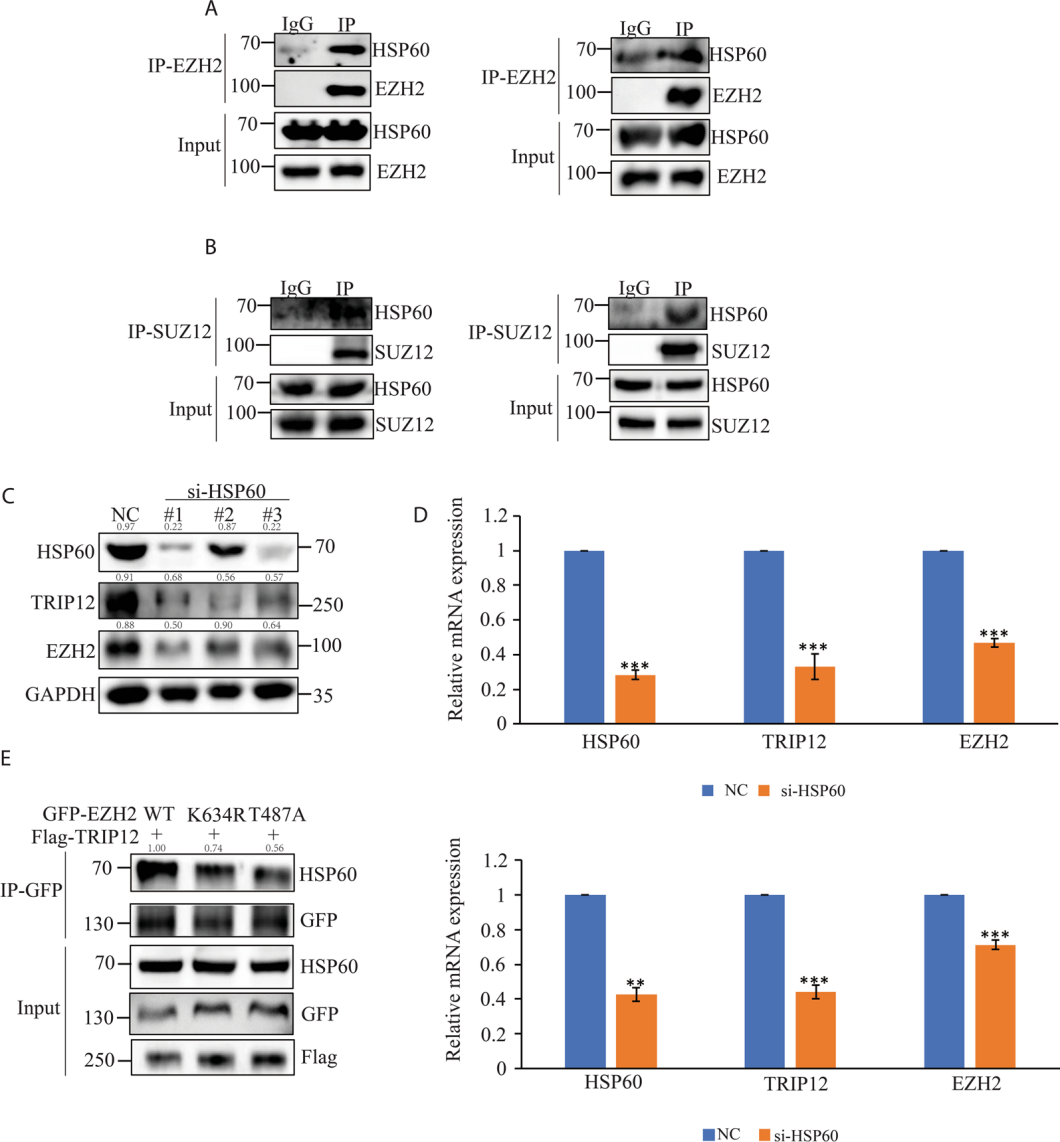
HSP60 is an upstream modulator of TRIP12-EZH2 signaling. **A** Co-IP showing interaction between HSP60 and EZH2 in YT (left) and NKYS (right) cells. **B** Co-IP showing interaction between HSP60 and SUZ12 in YT (left) and NKYS (right) cells. **C** Immunoblots indicating decrease of both TRIP12 and EZH2 with siRNA-mediated knockdown of HSP60 in NK92 cells. **D** Change of TRIP12 and EZH2 mRNA level with TRIP12 knockdown in YT (upper) and NKYS (lower) cells. **E** Change of interaction between HSP60 and EZH2 or its mutants with TRIP12 overexpression in HEK293T cells. N = 3 individual experiments and representative images are shown

Heat shock family proteins (HSPs) were indicated by the MS data as interacting partners of EZH2. And robust interactions were validated between HSP60 and EZH2 (Fig. 6A, Additional file 1: Fig. 2B), SUZ12 (Fig. 6B) or TRIP12 (Additional file 1: Fig. 2C) but not HSP90 and PRC2 subunits (Additional file 1: Fig. 2A) in ENKTL cells. When HSP60 was knocked down, a decrease in the TRIP12 and EZH2 protein and mRNA levels were noted (Fig. 6C, D), and conversely, when TRIP12 was knocked down there was no change in HSP60 level (Additional file 1: Fig. 2D), implying HSP60 was an upstream modulator of TRIP12 and EZH2. The interaction between HSP60 and EZH2 was hampered when the K634 ubiquitination site or the T487 phosphorylation site was mutated (Fig. 6E), hinting that HSP60 might also participate in mediating EZH2 K634 ubiquitination and T487 phosphorylation like TRIP12. Conventionally, HSP60 mainly locates within the mitochondria to act as a chaperone [23]. Unexpectedly in ENKTL cells, HSP60 also localized in the cytoplasm along with TRIP12 and EZH2 (Additional file 1: Fig. 2E), suggesting mitochondrial HSP60 might somehow translocate to cytoplasm for an interplay with TRIP12 and EZH2. Similar to TRIP12 and EZH2, HSP60 expression positively mediated ENKTL cell survival (Additional file 1: Fig. 3A and B), and manipulation of HSP60 level regulated canonically-repressed and noncanonically-activated target genes of EZH2 in ENKTL (Additional file 1: Fig. 3C–F). All the results above highlight the role of HSP60 as an upstream mediator of TRIP12-EZH2 signaling in ENKTL, which may translocate from mitochondria to co-locate with TRIP12 and EZH2 and promote EZH2 T487 phosphorylation (Additional file 2).

### Steroid therapy effectively harnesses abnormally active TRIP12-EZH2 signaling

**Fig. 7:**
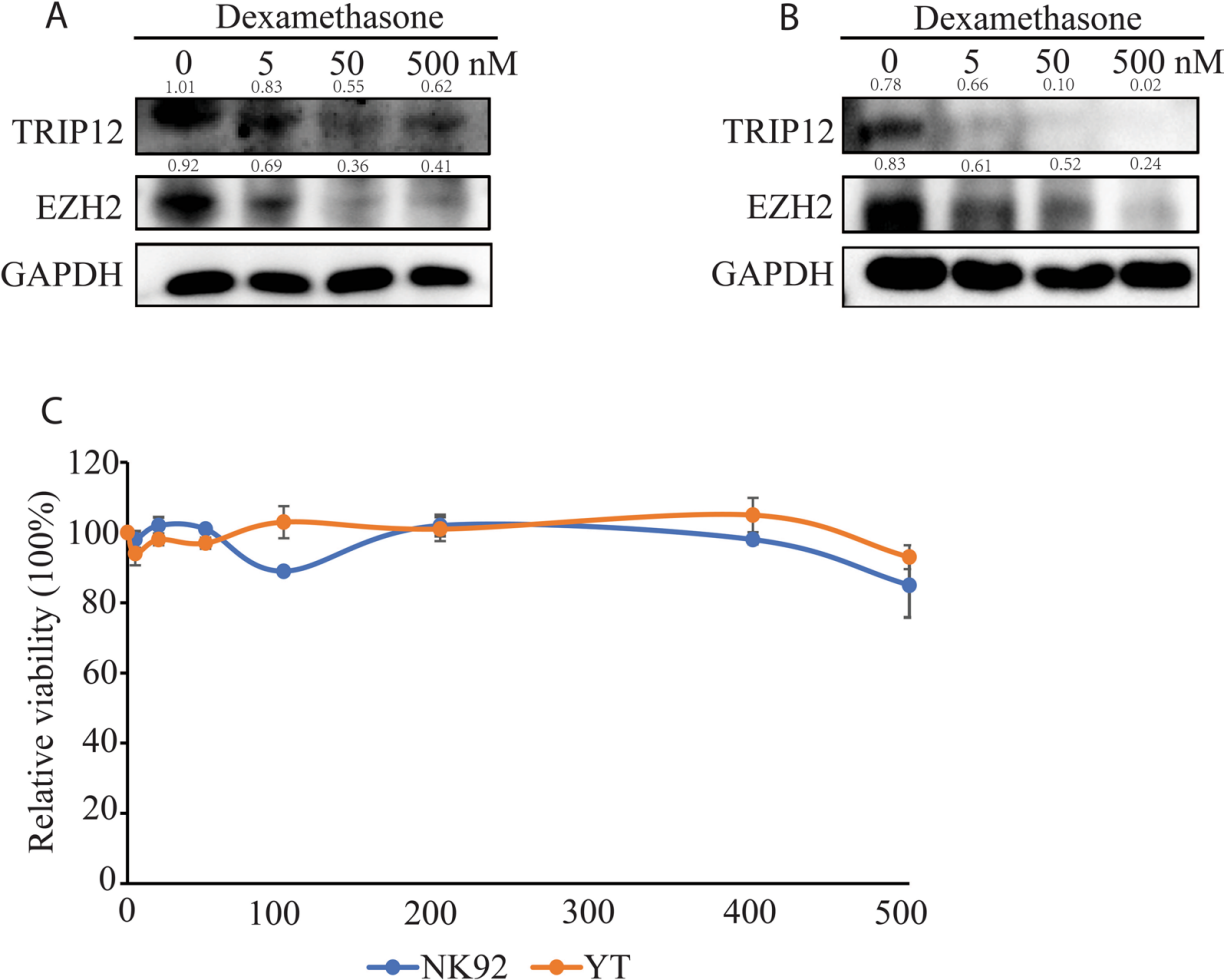
Dexamethasone effectively targets the TRIP12-EZH2 cascade. (**A**, **B**) Immunoblots showing change of TRIP12 and EZH2 level upon dexamethasone treatment with indicated concentrations in NK92 (**A**) and YT (**B**). N = 3 individual experiments and representative images are shown. **C** Relative viability of ENKTL cells upon dexamethasone treatment for 48 h. Cell Counting Kit-8 assay was used to measure cell viability

Corticosteroids have been used for a long time as an indispensable component in anti-neoplasm prescriptions including anti-ENKTL regimens. As reported recently, IDO is able to degrade tryptophan which modifies TRIP12 at post-translational level, stabilizes TRIP12 and promotes its E3 ligase activity [19]. Naturally, IDO activation may suppress TRIP12-EZH2 signaling. As expected, steroid agonist of IDO dexamethasone reduced the level of TRIP12 and EZH2 in ENKTL cell lines (Fig. 7A, B) without notably affecting cell survival (Fig. 7C). These findings suggest an unexpected role of clinically-available therapeutics corticosteroids in degrading EZH2 in ENKTL.

### The TRIP12-EZH2 cascade promotes ENKTL metastasis

**Fig. 8:**
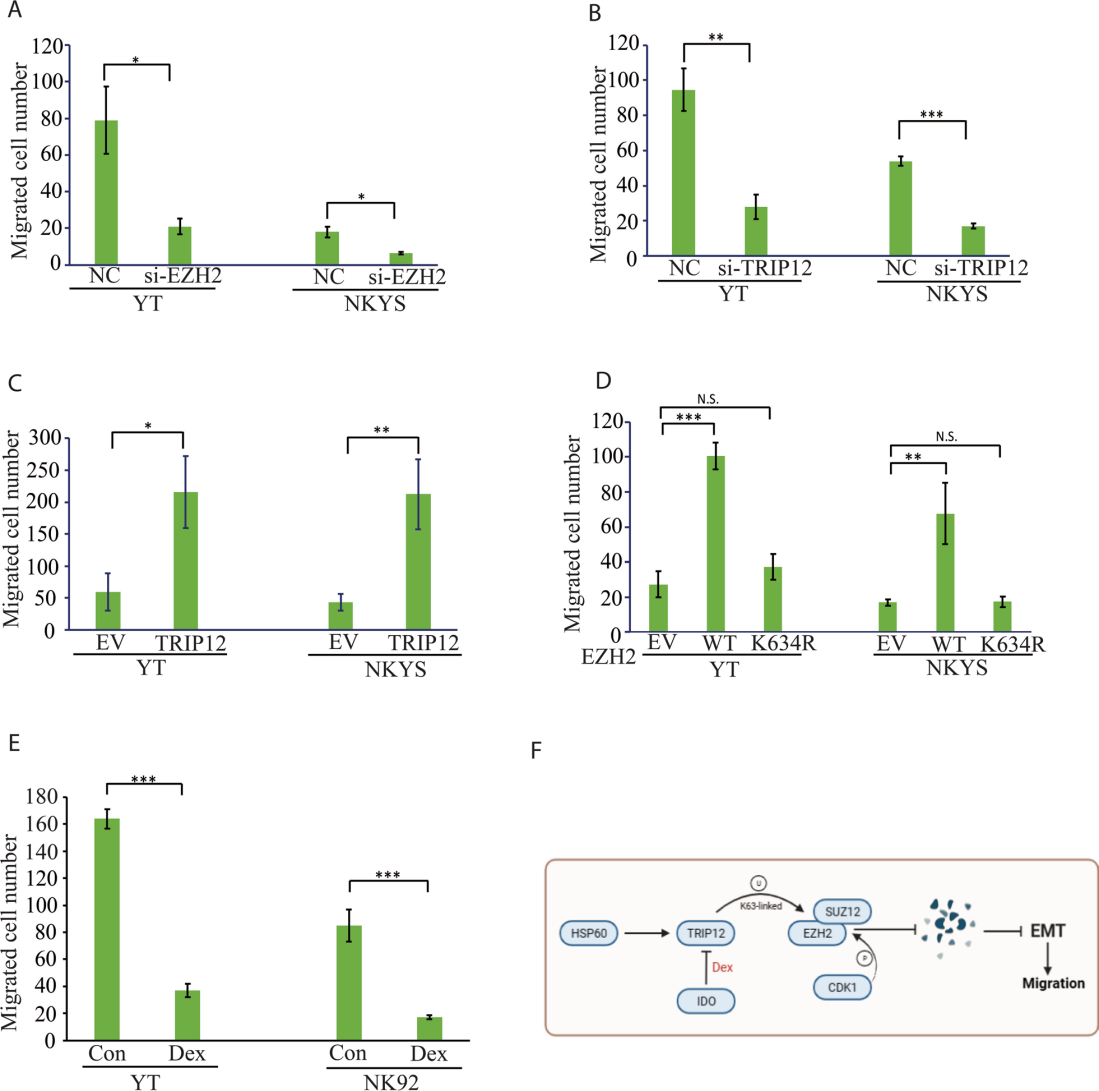
TRIP12-EZH2 axis regulates ENKTL cell migration. **A**–**E** The number of migrated NKTL cells to the lower compartment of transwell chamber with indicated transfections (**A**–**D**) or dexamethasone treatment (**E**, 48 h, 500 nM). The cells were subjected to transwell assay 16 h (**A**, **B**) or 24 h (**C**, **D**) after electroporation. N = 3; N.S. not significant; *p < 0.05; **p < 0.01; ***p < 0.001. **F** Schematic diagram illustrating the TRIP12-EZH2 signaling cascade

EMT is a key mechanism underpinning tumor metastasis, and during this process the level of epithelial marker E-CAD is down-regulated along with the up-regulation of transcription factors such as SNAIL and SLUG [24]. As stated above, EZH2 may accelerate cancer metastasis by directly participating in EMT, and therefore, in this study we further examined the role of TRIP12-EZH2 signaling axis in regulating EMT. Knockdown of EZH2 and TRIP12 both attenuated the capacity of ENKTL cell migration (Fig. 8A, B) in line with an induction of E-CAD and a reduction of SNAIL and SLUG (Additional file 1: Fig. 4A and B). A conversed phenomenon was observed with TRIP12 overexpression (Fig. 8C, Additional file 1: Fig. 4C and D). Overexpression of EZH2 WT greatly enhanced ENKTL cell migration and modulated EMT biomarker expression profile similar to TRIP12 overexpression, and notably this could be rescued by EZH2 K634R mutant transfection (Fig. 8D, Additional file 1: Fig. 4E and F). Moreover, dexamethasone treatment repressed ENKTL cell migration as expected (Fig. 8E). Our findings suggest the TRIP12-EZH2 signaling facilitates EMT and migration of ENKTL cells and dexamethasone treatment nicely suppressed ENKTL migration via degrading TRIP12-EZH2.

## Discussion

The mechanisms directly or indirectly rendering EZH2 overexpression has not been fully clarified in ENKTL, and all those commercially-available EZH2 inhibitors, no matter in the form of small-molecule [12, 13, 15] or peptide [14], cannot target the H3K27 tri-methylation and PRC2 independent oncogenic functions of EZH2. Therefore, it is urgent to further examine the mediation of oncogenic EZH2 in this lymphoid malignancy. This study demonstrates that the E3 ligase TRIP12 triggers K63-linked polyubiquitination of EZH2 at K634 for stabilization and promotes the EMT process and cell migration in ENKTL. Our results uncover a previously undefined mechanism of EZH2 turnover via TRIP12-modulated polyubiquitination. Furthermore, EZH2 enhances EMT and cell migration resultantly, which might be further intensified by TRIP12 overexpression through EZH2 stabilization.

In most situations, EZH2 exhibits carcinogenic effects via H3K27 tri-methylation which turns off the expression of tumor suppressive genes. Thus, the tumorigenic activity of EZH2 could be block by small-molecule inhibitors like GSK126. However, in several other cancer types like ENKTL, EZH2-mediated oncogenesis is enzymatically- and PRC2-independent, and naturally, inhibitors aiming at enzymatic disruption are ineffective to target oncogenic EZH2. Several new strategies have been adopted in recent years to harness pan-EZH2, including EZH2 degrader MS1943 [15] and EZH2 proteolysis target chimeras (PROTACs) [25–27]. Although these compounds have been shown to specifically degrade EZH2 in a few malignancies like triple negative breast cancer [26, 27] and diffuse large B cell lymphoma [25], few report has indicated their effectiveness in ENKTL. We have demonstrated that MELK stabilized EZH2 by catalyzing phosphorylation followed by deubiquitination in ENKTL [8]. However, some of commercialized MELK inhibitors like OTSSP167, are still controversial in that the tumoricidal effects might not be due to enzymatic inhibition of MELK [28]. Therefore, more studies are needed to further examine other regulators of EZH2 turnover in ENKTL.

Overexpressed EZH2 also participates in mediating EMT, one of the major factors contributing to cancer metastasis, besides conferring survival advantages. During EMT process, epithelial cells no longer stick with each other, get out of the extracellular matrix, acquire mesenchymal properties and move to distant tissues or organs. EZH2 overexpression has been linked to the downregulation of epithelial markers, upregulation of mesenchymal counterparts and EMT-associated transcriptional factors in a variety of cancers [1, 29, 30]. Post-translational modifications, like methylation, have been reported to affect the behaviors of EZH2 in mediating EMT [1]. ENKTL is one of the most lethal lymphoid malignancies with extremely poor prognosis especially in advanced cases. Although metastasis is often seen in advanced ENKTL that may involve masculine organ and central nervous system [31, 32], currently very few studies have explored the underlying mechanisms rendering ENKTL metastasis.

TRIP12 is a HECT family E3 ligase and regulates important biological processes such as cell cycle progression, DNA damage repair, chromatin remodeling and cell differentiation by mediating the turnover of substrate proteins [33]. In most cases, TRIP12 as an E3 ligase directly attaches ubiquitin chain to the substrates and initiates proteasome-mediated degradation [17, 34]. This E3 ligase may inhibit EMT process in breast cancer as reported before [35]. In this study, we reveal a new and distinct role of TRIP12 in stabilizing the substrate protein EZH2 via K63-linked polyubiquitination to promote EMT and metastasis in ENKTL. TRIP12 inhibition for EZH2 degradation might be beneficial to overcome ENKTL metastasis. Until today, no commercialized TRIP12 inhibitor is available for preclinical studies as the three-dimensional structure of TRIP12 is still unknown. This study has revealed dexamethasone treatment effectively blocked TRIP12-EZH2 signaling possibly by IDO activation. Although dexamethasone is part of existing regimens for ENKTL, our study illustrates a novel mechanism further explaining how dexamethasone acts to benefit ENKTL patients.

The group I chaperone HSP60 mostly resides in mitochondria to maintain protein homeostasis and may transfer to other intracellular or even extracellular locations for a couple of reasons, depending on which interactor is there [18]. Cytosolic HSP60, unlike the mitochondrial HSP60, has a role in mediating tumor cell apoptosis [18]. Our study finds out that HSP60 translocates to cytosol, interacts with PRC2 components and regulates TRIP12-EZH2 signaling in ENKTL. These results identify a new signaling axis and enrich the functional connotation of cytosolic HSP60.

## Conclusion

Altogether, in this study we demonstrate that in ENKTL, TRIP12 stabilizes EZH2 through site-specific K63-linked ubiquitination thereby enhancing PRC2 cohesion and CDK1-catalyzed EZH2 phosphorylation, which is regulated by cytosolic HSP60. This promotes ENKTL cell migration via EMT-related mechanisms, which could be harnessed by corticosteroid therapy (Fig. 8F).

## Materials and methods

### Cell culture

The ENKTL cell lines used in this study included NKYS, YT and NK-92. NKYS cells were cultured in RPMI-1640 (Gibco) supplemented with 10% fetal bovine serum (Gibco) and 300 U/mL of interleukin-2 (Miltenyi). YT cells were cultured in Iscove's Modified Dulbecco Medium (Gibco) supplemented with 20% fetal bovine serum (Gibco), 1% Sodium Pyruvate (Gibco) and 100 U/mL of interleukin-2 (Miltenyi). NK-92 cells were cultured in RPMI-1640 (Gibco) supplemented with 10% fetal bovine serum (Gibco), 10% horse serum (Bioagrio) and 100 U/mL of interleukin-2 (Miltenyi). In addition, HEK293T cells were cultured in Dulbecco’s modified Eagle medium (Gibco) supplemented with 10% fetal bovine serum (Gibco).

### Antibodies and drug

Primary antibodies and drugs used in this study include: SUZ12 (Santa Cruz sc-271325, 1:1000), EED (Santa Cruz sc-518116, 1:1000), normal mouse IgG (Santa Cruz sc-2025), Flag (Beyotime AF519, 1:1000), HSP60 (Santa Cruz sc-13115, 1:1000), EZH2 (Cell Signaling Technology 3147 s, 1:1000), Anti-Ubiquitin (linkage-specific K63) (Abcam EPR8590-448, 1:1000), GAPDH (Santa Cruz sc-47724, 1:1000), Tri-Methyl-Histone H3 (Lys27) (Cell Signaling Technology C36B11, 1:1000), Histone H3 (Cell Signaling Technology D1H2, 1:2000), UHRF1 (Santa Cruz sc-373750, 1:1000), TRIP12 (proteintech 25303-1-AP, 1:1000), β-tubulin (Wanleibio WL01931, 1:750), GFP (Santa Cruz sc-9996, 1:1000), HA-probe (Santa Cruz sc-7392, 1:1000), HSP90 (Beyotime AH732, 1:1000), COX-IV (Wanleibio WL02203, 1:1000), CDK1 (Santa Cruz sc-54, 1:1000), EZH2 (Phospho-Thr487) (Signalway #12820, 1:1000), MG132 (Targetmol, 133407-82-6) and dexamethasone (Targetmol, T1076/50-02-2).

### Constructs and siRNA transfection

For ENKTL cell lines, transfections with constructs and small interfering RNAs (siRNAs) were performed using the Neon Transfection system (Invitrogen) according to the manufacturer’s instructions. For HEK293T cells, the LIPOFECTAMINE3000 (Invitrogen) was used for constructs transfections.

The pcDNA3.1-Flag-TRIP12, pcDNA3.1-Myc-His-HSP60 and pcDNA empty vector were purchased from Public Protein/Plasmid Library. The pReceiver-M98-GFP-EZH2, pReceiver-M98 empty vector, human TRIP12 siRNA and EZH2 siRNA were purchased from Shenggong. The Prk5-HA-Ub, Prk5-HA-K11-Ub, Prk5-HA-K48-Ub and Prk5-HA-K63-Ub plasmids were kind gifts from Prof Hong Zhu (Zhejiang University). The Human HSP60 siRNA were purchased from Qingke. Sequences of siRNAs are indicated in Table 2.

**Table 2:** siRNA sequences

Gene	SiRNA
EZH2	GACUCUGAAUGCAGUUGCU
TRIP12	CCCUCAAGGUCGAUUAAGUTT (#1)
	CGAGCCUUAACAUACAUGATT (#2)
	GACCGAGCAAUGCAAAGAUTT (#3)
HSP60	GCACAGGUUGCUACGAUUU (#1)
	GCUUCAAGGUGUAGACCUU (#2)
	CAAUGACCAUUGCUAAGAA (#3)

### Cell survival assay

The cell survival was measured with the Cell Counting Kit-8 (Beyotime) according to the manufacturer’s indications.

### Mitochondrial and plasma protein separation

The Mitochondrial and Plasma Protein Separation Kit (Beyotime) was used for the fractionation based on the manufacturer’s protocols. Both the cytoplasmic and Mitochondrial lysates were used for immunoblots.

### Co-immunoprecipitation (Co-IP)

Co-IP experiments were performed using HEK293T and ENKTL cells. The cells were lysed with RIPA buffer supplemented with PMSF, which were subject to sonication (Ultrasonic Cell Crusher Noise Isolating chamber) later. After centrifugation for clarification, antibodies were added in to the cell lysates and the lysates were shaken at 4 °C overnight. The protein A/G beads (invitrogen 10004D) were then added, followed by rotation and incubation at 4 °C for 1 h. Then the beads were washed sequentially with lysis buffer and PBS, and 5 × SDS loading buffer (Ding Guo) was added to the beads, which were then heated at 100 °C for 5 min before loading for immunoblots.

### Immunoblot

Total protein was separated by SDS-PAGE with different gel concentrations and transferred onto PVDF membranes. Membranes were blocked in 5% skim milk in PBS with Tween-20 (PBST) for 2 h before incubation with 1:1000 diluted primary antibody at 4 °C overnight. Membranes were then washed with PBST for 5 times and incubated with 1:5000 diluted secondary antibody for 2 h. The signals of the proteins on the membrane were visualized through Tanon Imaging System and analyzed with ImageJ software.

### Cell migration assay

5 × 10^4^ ENKTL cells was dispersed in 100 μL serum-free medium and seeded in the upper compartment of the transwell chambers with 500 μL medium supplemented with 10% fetal bovine serum and 100 U/mL of interleukin-2 in the transwell lower compartment and incubated at 37 °C for 12 h. The migrating cells located in the lower compartment of transwell chambers were counted under microscope. A total of 3 views of fields were randomly chosen, photographed and counted.

### Quantitative real-time PCR (qRT-PCR)

Total RNA was extracted using RNAsimple Kit (Tiangen, DP419) and reverse-transcribed with the Fastking RT kit (Tiangen). The reaction was performed on a ConnectTM Real-Time System (CFX) with SYBR Green Mix (Tiangen). The primers used for qRT-PCR are indicated in Additional file 1.

### Site-directed mutagenesis

EZH2 K61R, K602R, K634R, K735R and T487A were generated through mutagenesis using the Fast Mutagenesis System (TransGen Biotech FM111-02) following the manufactures’ protocols. The primers used for generating the EZH2 mutants are shown in Additional file 1. The EZH2 K569R plasmid was generated through cloning.

### Gene expression profiling (GEP) and data analysis

Gene expression profiling (GEP) has been performed on patient samples from 35 ENKTL cases using a GeneChip® Clariom D Assay (Human) array, and raw data have been uploaded as described before (GSE160119) [36]. The analysis and visualization of gene expression data were performed using R (v4.2). Gene expression data were first normalized with the robust multi-array average (RMA) algorithm through oligo package (v1.48.0). The expression correlation between TRIP12 and EZH2 was evaluated by Spearman's method, and the comparison of the EZH2 expression between ENKTL and normal samples was performed by Mann–Whitney U test with ggplot2 (v3.3.6) and ggpubr (v0.4.0) packages.

### Liquid chromatography-mass spectrometry-mass spectrometry (LC–MS/MS) analysis

HEK293T cells in culture were subjected to transfection, followed by cell harvesting and lysis. And co-IP was performed using the lysate with EZH2 pull-down until beads washing. Then 50 mM ammonium bicarbonate was added to the beads to a final volume of 100 μL. And the samples were then reduced by DTT for 1 h at 56 °C and alkylated by IAA for 1 h at room temperature in dark. Trypsin was added in at a ratio of 1:50, and the mixtures were incubated at 37 °C for digestion overnight. Afterwards, the solutions were lyophilized and the extracted peptides were dried.

Before analysis, the peptides in dryness were reconstituted in 0.1% formic acid. The LC–MS/MS analysis was performed on an Easy-nLC1200 System coupled with a Q Exactive™ Hybrid Quadrupole-Orbitrap™ Mass Spectrometer (Thermo Fisher). 5 μL of each sample was loaded onto C18 PepMap100 trap column and eluted on an Acclaim PepMap RPLC analytic column. A 60 min gradient procedure for each single analysis was performed as follows: 4–8% B for 2 min, 8–28% B for 43 min, 28–40% B for 10 min, 40–95% B for 1 min and 95–95% B for 10 min (A = 0.1% formic acid in water, B = 0.1% formic acid in water-acetonitrile (1:4)). The flow rate was set at 0.6 μL/min. Data-dependent mode was chosen for the MS with a full scan (300–1800 m/z) and 3 s per cycle was set. High energy collision dissociation was used to perform peptide fragmentation. Then the MS spectra were acquired at the resolution of 70,000 with a maximum integration time of 100 ms.

Next, the raw MS files were analyzed and searched against protein database based on the homo sapiens species using MaxQuant (1.6.2.10). The mass tolerance was set to 20 ppm for both the precursor and the fragment ion, with two missed cleavages allowed.

### Supplementary Information

**Additional file 1**. Supplementary Information-clinical epigenetics.

**Additional file 2**. Original blot.

## Data Availability

Raw data of the GEP analysis were previously uploaded to the Gene Expression Omnibus (GSE160119). Raw data of the LC–MS/MS analysis has been uploaded to proteomeXchange Consortium via PRIDE (PXD046145).

## Abbreviations

co-IP: Co-immunoprecipitation
EMT: Epithelial-mesenchymal transition
ENKTL: Extranodal natural killer/T cell lymphoma
GEP: Gene expression profiling
HSP: Heat shock protein
LC–MS/MS: Liquid chromatography-mass spectrometry-mass spectrometry
MS: Mass spectrometry
PRC2: Polycomb repressive complex 2
PROTACs: Proteolysis target chimeras
qRT-PCR: Quantitative real-time PCR
siRNAs: Small interfering RNAs
UPS: Ubiquitin-proteasome system
WT: Wildtype
