# Supplementary material for: EZH2 K63-polyubiquitination affecting migration in extranodal natural killer/T-cell lymphoma
Source: Clin Epigenetics. 2023 Nov 29;15:187. doi: 10.1186/s13148-023-01606-6 (PMC10685657; doi:10.1186/s13148-023-01606-6)
Supplement: Supplementary file 1 — Additional file 1. Supplementary Information-clinical epigenetics. [file 13148_2023_1606_MOESM1_ESM.docx]

**Supplementary Information**

**EZH2 K63-polyubiquitination affecting migration in extranodal natural killer/T-cell lymphoma**

**1 Supplementary Materials and Methods**

**1.1 Quantitative real-time PCR (qRT-PCR)**

The qRT-PCR experiments were performed as described in the main text. The primers used are summarized in Supplementary table 1.

| Supplementary table 1. Primers for qRT-PCR | |
| --- | --- |
| Gene | Primer |
| EZH2 | 5’-AAGCACAGTGCAACACCAAG-3' (forward) |
|  | 5’-CAGATGGTGCCAGCAATAGA-3' (reverse) |
| TRIP12 | 5’- TTCACAGAGGAACACTGCCG-3' (forward) |
|  | 5’- GCCTGCCCTAAATGTGACCT-3' (reverse) |
| MYT-1 | 5’- ACAAAGGCAGATACCCAACG-3' (forward) |
|  | 5’-GCAGTTTCAAAAAGCCATCC-3' (reverse) |
| SLIT-2 | 5’-CCAAGTTCATCCTTGGGAGA-3' (forward) |
|  | 5’-AAGGCAGTAGAGCCCACTCA-3' (reverse) |
| SOX17 | 5’-AATCGCTAGGCCGATTTCTT-3' (forward) |
|  | 5’-GAAGGGAACCCAGAGCCTTA-3' (reverse) |
| CCND1 | 5’-CCGATGCCAACCTCCTCAACGA-3' (forward) |
|  | 5’-CATCCAGGTGGCGACGATCTTCC-3' (reverse) |
| RAD51C | 5’-GCAGCCGAGTTTTCTCATTC-3' (forward) |
|  | 5’-GAGACGCTGTTGGTCCTGAT-3' (reverse) |
| KRAS | 5’-CGCGGCGTCTCCCTGGTG-3' (forward) |
|  | 5’-CTTGGCGGGAGGAGGGACTGC-3' (reverse) |
| MAPK15 | 5’-TGCCGAGGGAGGTGCTTG-3' (forward) |
|  | 5’-ACGCTGCTCTCCTGTCCCTGT-3' (reverse) |
| SNAIL | 5'-AAGATGCACATCCGAAGCCA-3' (forward) |
|  | 5'- CATTCGGGAGAAGGTCCGAG-3' (reverse) |
| SLUG | 5'-GCTACCCAATGGCCTCTCTC-3' (forward) |
|  | 5'-CTTCAATGGCATGGGGGTCT-3' (reverse) |
| E-CAD | 5'-GAAACTGGCATCCTCACAGC-3' (forward) |
|  | 5'-TACTGCTGCTTGGCCTCAAA-3'(reverse) |
| HSP60 | 5'-CCGACGACCTGTCTCGCC-3' (forward) |
|  | 5'-TTGGCATAAGCCCGAGTGAG-3' (reverse) |

**1.2 Site-directed mutagenesis**

The qRT-PCR experiments were performed as described in the main text. The primers used for mutating EZH2 plasmids are shown in Supplementary table 2.

| Supplementary table 2. Mutagenesis primers | |
| --- | --- |
| Mutation | Primer |
| EZH2 K61R | 5‘-CTTAAACCAAGAATGGAGACAGCGAAGG-3’ (forward) |
|  | 5‘-CTCCATTCTTGGTTTAAGATTTCCGTTCTTTCC-3’ (reverse) |
| EZH2 K602R | 5‘-AAAATGTGTCCTGCAGGAACTGCAGT-3’ (forward) |
|  | 5‘-CTGCAGGACACATTTTTACTGTCCCA-3’ (reverse) |
| EZH2 K634R | 5‘-CAAAGATCCTGTGCAGAGAAATGAATTCATC-3’ (forward) |
|  | 5‘-CTCTGCACAGGATCTTTGATAAAAATCCCCC-3’ (reverse) |
| EZH2 K735R | 5‘-AGGCTGATGCCCTGAGGTATGTCGGC-3’ (forward) |
|  | 5‘-CTCAGGGCATCAGCCTGGCTGTATCT-3’ (reverse) |
| EZH2 T487A | 5‘-CTGAGGATGTGGATGCTCCTCCAAGGAA-3’ (forward) |
|  | 5‘-CATCCACATCCTCAGCGGGAGCTGGAGTCA-3’ (reverse) |

**2 Supplementary figures**


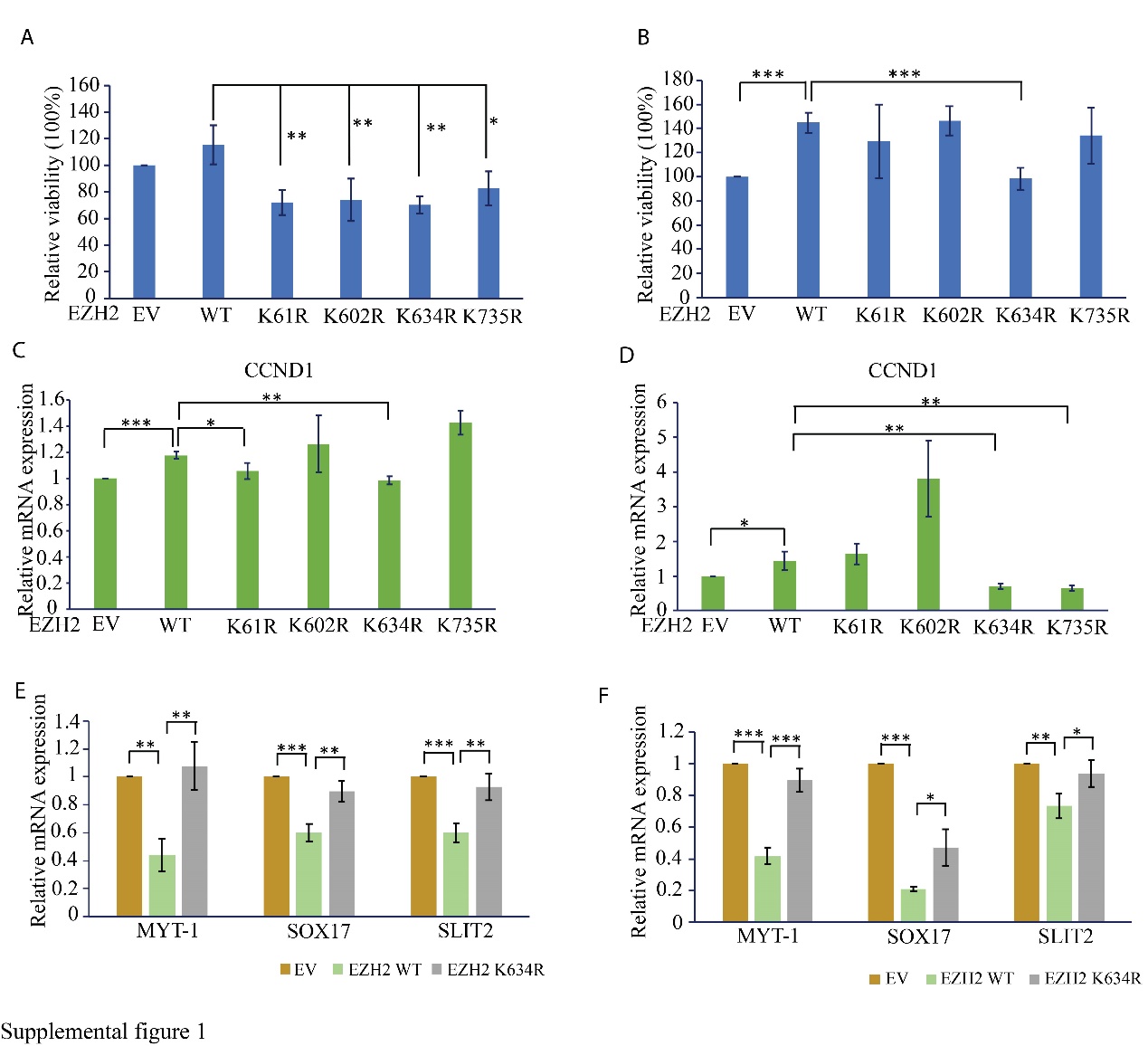


**Supplemental figure 1. Effects of ubiquitination-null mutants in mediating EZH2-related oncogenesis.** (A and B) Relative cell survival with indicated transfections of empty vector, EZH2 wild type or mutants in YT (A) and NKYS (B) cells. ENKTL cells were used for transfection via electroporation, followed by Cell Counting Kit-8 assay 16 h after transfection. (C and D) Relative mRNA expression level of CCND1 with indicated transfections of empty vector, EZH2 wild type or mutants in YT (C) and NKYS (D) cells. (E and F) Relative mRNA levels of canonically-repressed genes of EZH2 with indicated transfections of empty vector, EZH2 wild type or K634R mutant in YT (E) and NKYS (F). ENKTL cells were used for transfection via electroporation, and the cells were harvested for RNA extraction 16 h after transfection. Results are mean ± SD. N=3; **p*<0.05; ***p*<0.01; ****p*<0.001.


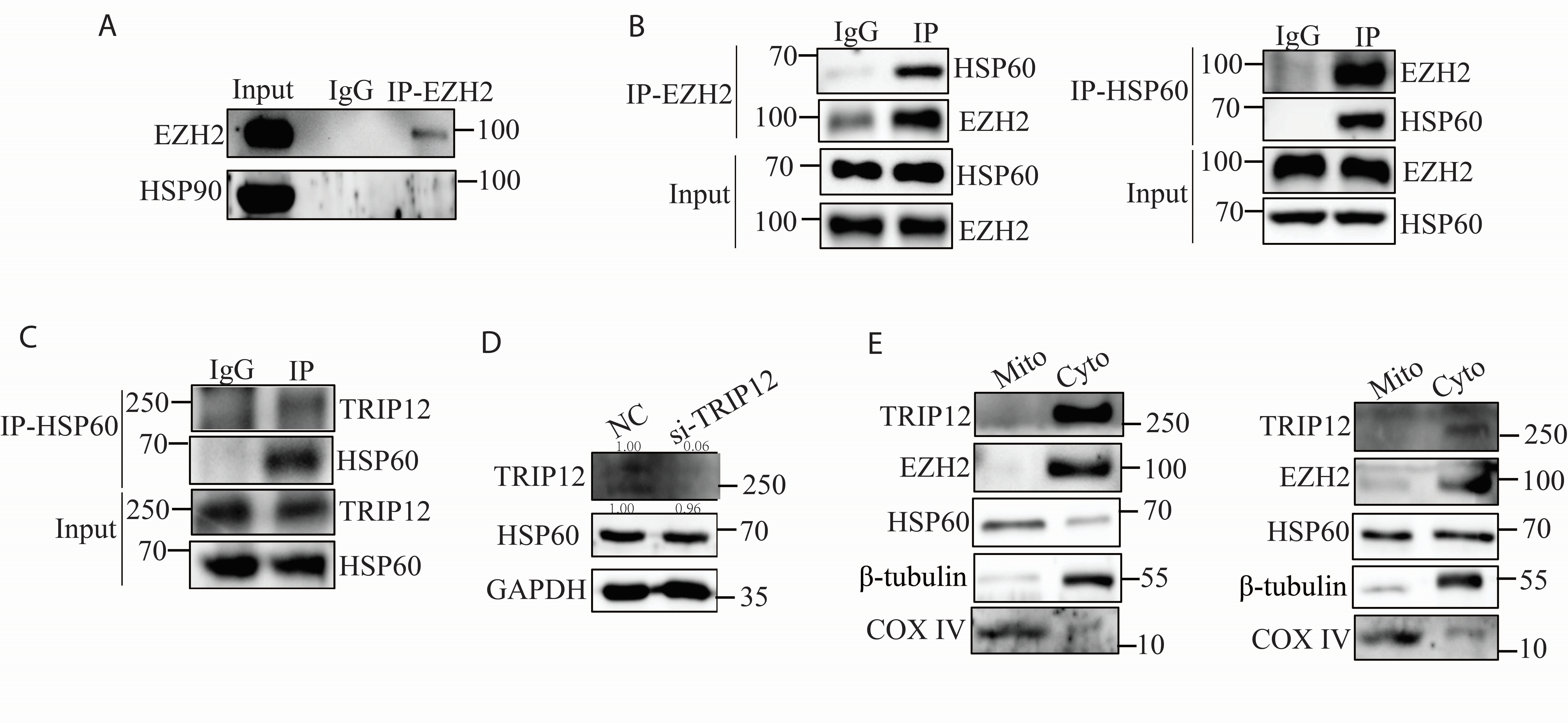


**Supplemental figure 2. HSP60 may mediate TRIP12-EZH2 cascade via a cytoplasmic role.** (A) No interaction was probed between EZH2 and HSP90 in YT cells. (B) Co-IP showing interaction between EZH2 and HSP60 in NK92 (left) and YT (right) cells. (C) Co-IP indicating interaction between TRIP12 and HSP60 in NK92 cells. (D) Si-RNA-mediated knockdown of TRIP12 did not affect HSP60 expression in YT cells. The cells were harvested for lysis and immunoblot 48 h after electroporation. (E) Mitochondria-cytoplasm fractionation indicating the subcellular localization of TRIP12, EZH2 and HSP60 in NK92 (left) and YT (right) cells. COX-IV and β-tubulin are markers for mitochondria and cytoplasm, respectively.


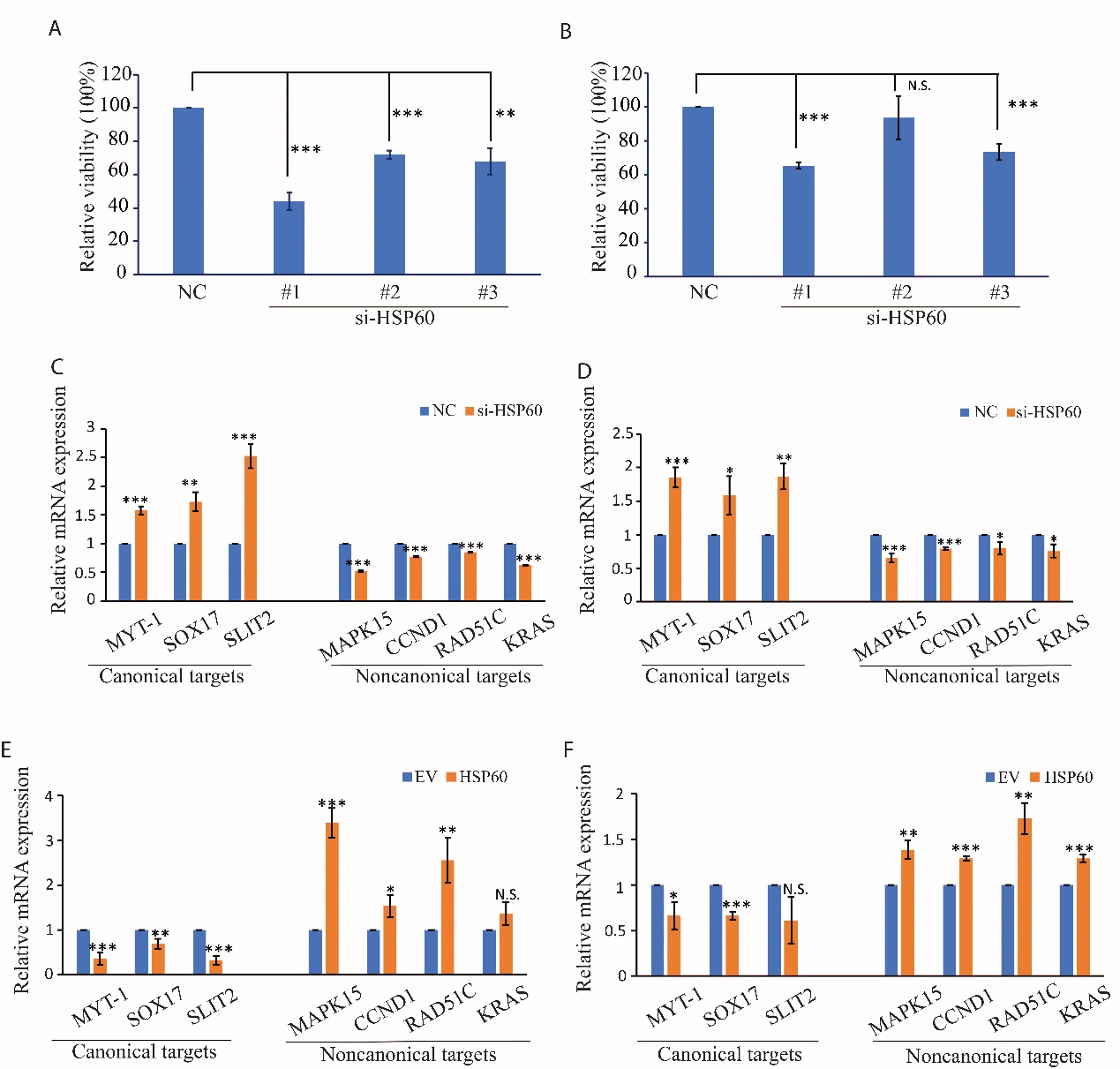


**Supplemental figure 3. HSP60 regulates ENKTL cell survival and EZH2 target gene expression.** (A and B) Relative viability with HSP60 knockdown in NKYS (A) and NK92 (B) cells. (C and D) Relative mRNA expression of canonically-repressed and noncanonically-activated target genes of EZH2 upon knockdown of HSP60 in NKYS (C) and NK92 (D). (E and F) Relative mRNA levels of canonically-repressed and noncanonically-activated genes of EZH2 with empty vector or HSP60 transfection in NK92 (E) and YT (F) cells. The cells were harvested for RNA extraction 16 h after electroporation. Results are mean ± SD. N=3; N.S. not significant; *p<0.05; **p<0.01; ***p<0.001.


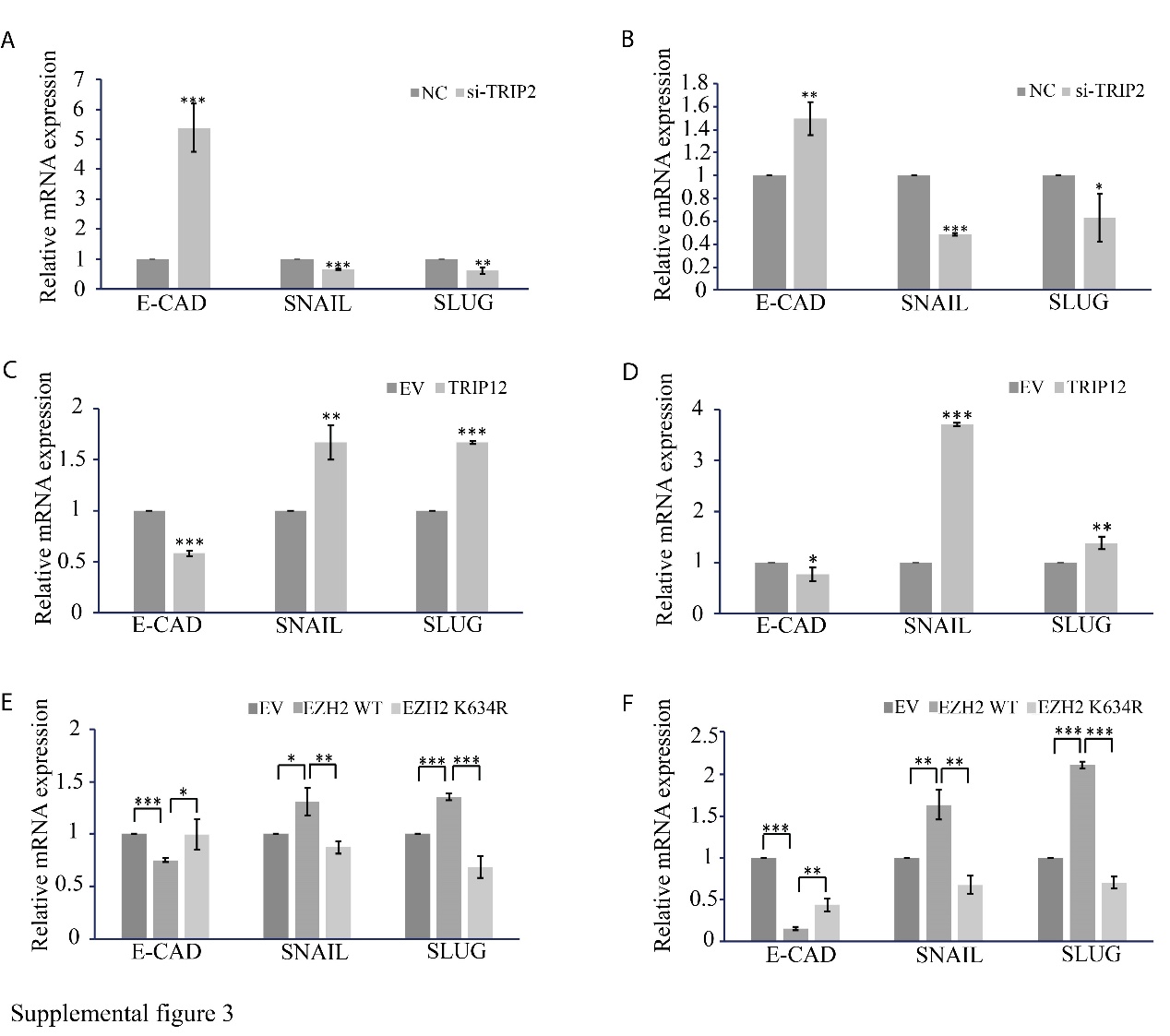


**Supplemental figure 4. The HSP60-TRIP12-EZH2 signaling axis modulates EMT processes in ENKTL.** (A-F) Relative mRNA levels of epithelial marker E-CAD and EMT-related transcription factors SNAIL and SLUG in YT (A, C and E) and NKYS (B, D and F) cells. The cells were harvested for RNA extraction 16 h (overexpression) or 24 hours (si-RNA-mediated knockdown) after electroporation. Results are mean ± SD. N=3; **p*<0.05; ***p*<0.01; ****p*<0.001.
