# Supplementary material for: EZH2 K63-polyubiquitination affecting migration in extranodal natural killer/T-cell lymphoma
Source: Clin Epigenetics. 2023 Nov 29;15:187. doi: 10.1186/s13148-023-01606-6 (PMC10685657; doi:10.1186/s13148-023-01606-6)
Supplement: Supplementary file 2 — Additional file 2. Original blot. [file 13148_2023_1606_MOESM2_ESM.pdf]

Original blot

EZH2 K63-polyubiquitination affecting migration in extranodal natural killer/T-cell lymphoma

Figure 1B WB

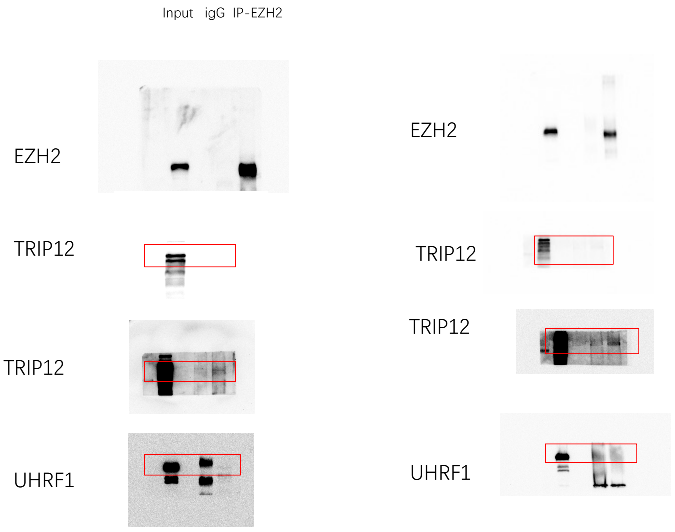

Figure 1C WB

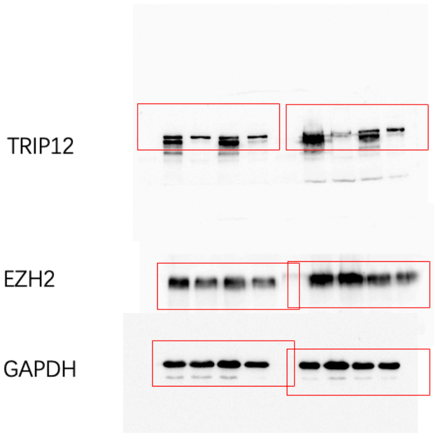

Figure 4A WB

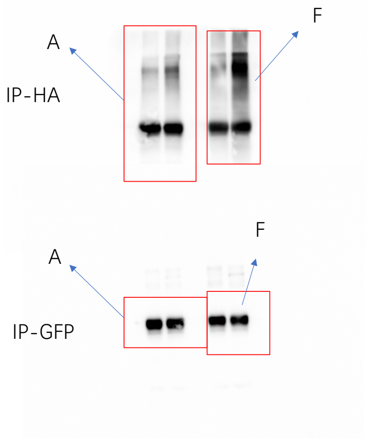

Figure 4F WB

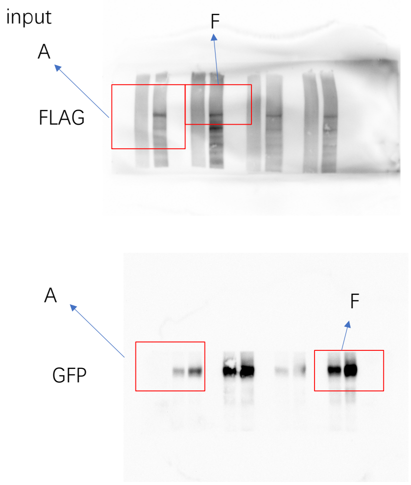

Figure 4D WB

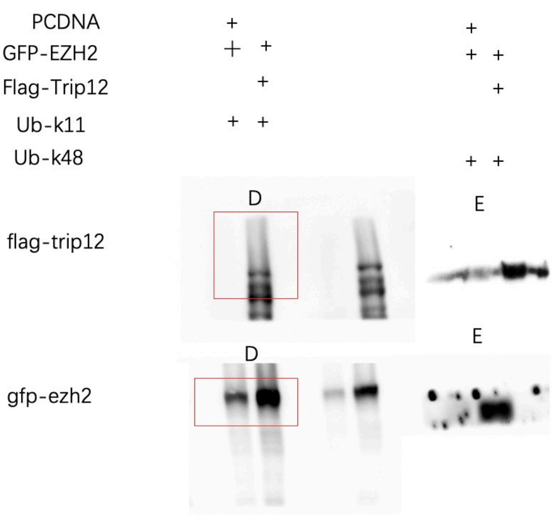

Figure 4E WB

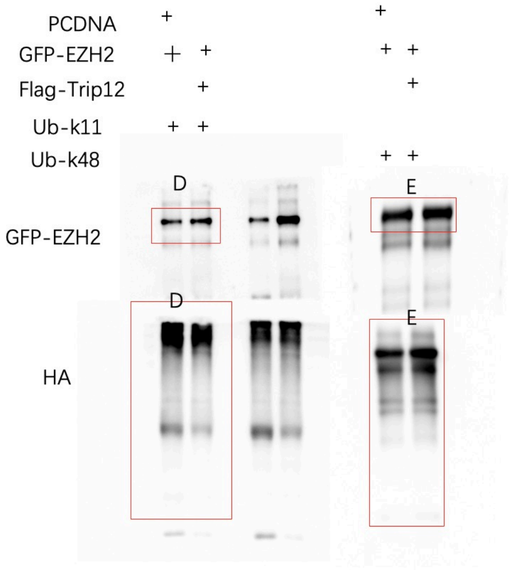

Figure 4G WB

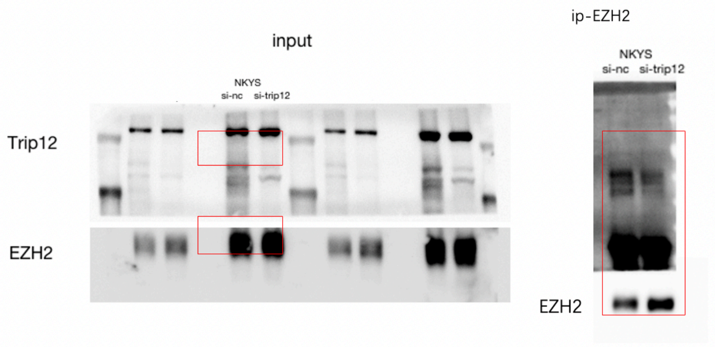

Figure 5A WB

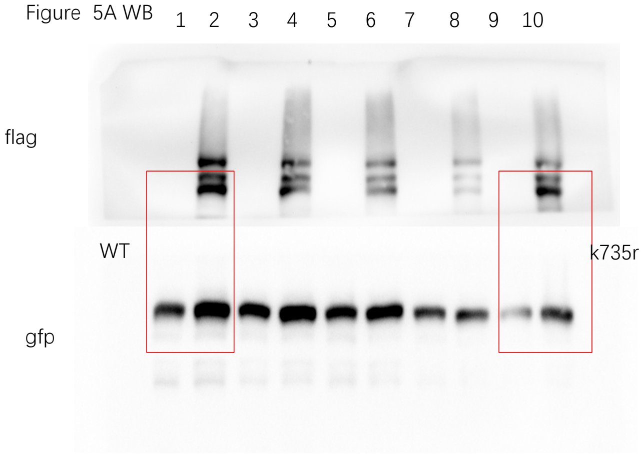

1 GFP-EZH2+PCDNA+K63-Ub
2 GFP-EZH2+Flag-Trip12+k63-ub
3 k61r+PCDNA+K63-Ub
4 k61r+Flag-Trip12+k63-ub
5 k602r+PCDNA+K63-Ub
6 k602r+Flag-Trip12+k63-ub
7 k634r+PCDNA+K63-Ub
8 k634r+Flag-Trip12+k63-ub
9 k735r+PCDNA+K63-Ub
10 k735r+Flag-Trip12+k63-ub

Figure 5A WB

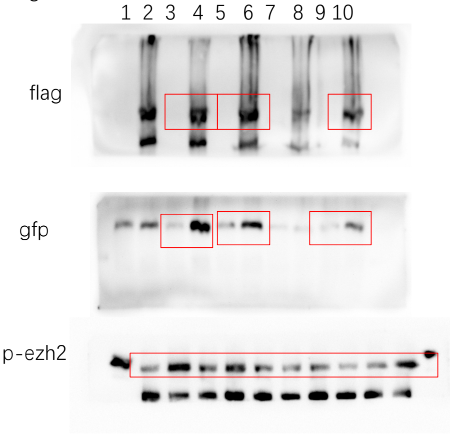

1 GFP-EZH2+PCDNA
2 GFP-EZH2+Flag-Trip12
3 k61r+PCDNA
4 k61r+Flag-Trip12
5 k602r+PCDNA
6 k602r+Flag-Trip12
7 k634r+PCDNA
8 k634r+Flag-Trip12
9 k735r+PCDNA
10 k735r+Flag-Trip12

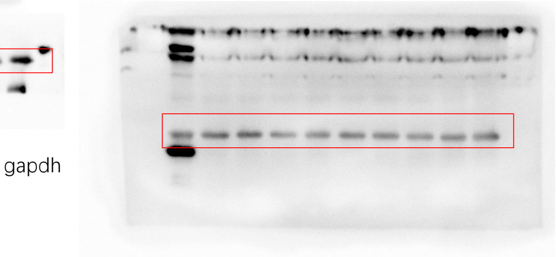

Figure 5A WB K569R

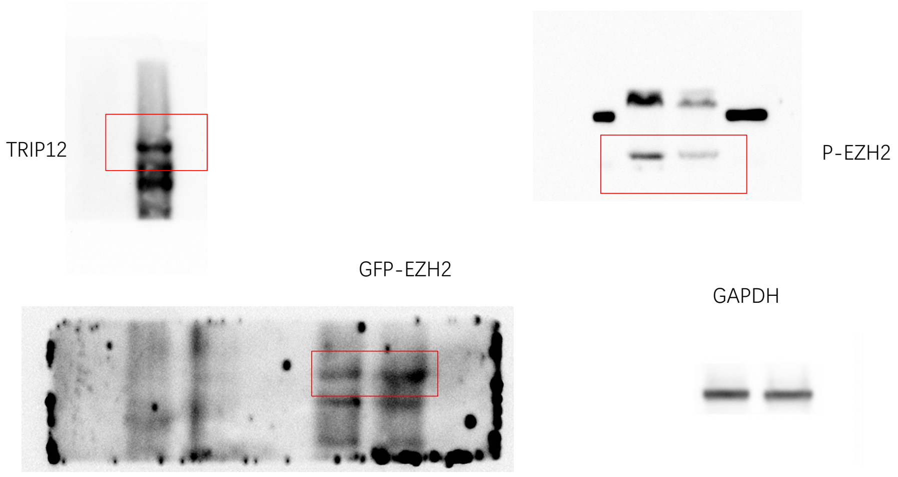

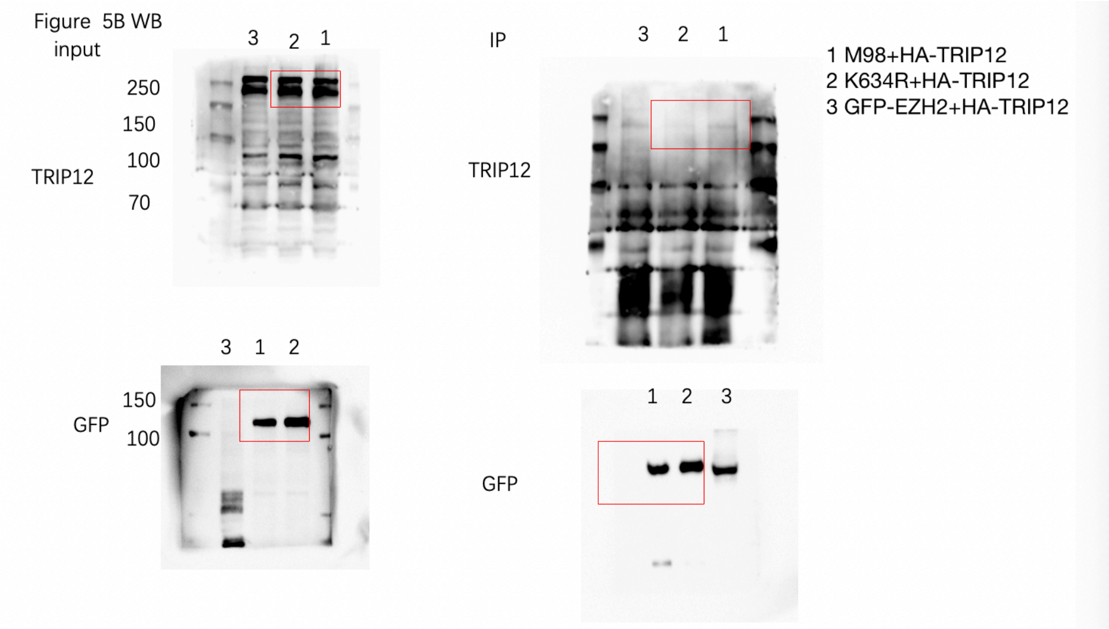
Figure 5B WB
input
3 2 1
250
150
100
70
TRIP12
3 1 2
150
100
GFP
IP
3 2 1
TRIP12
1 2 3
GFP
1 M98+HA-TRIP12
2 K634R+HA-TRIP12
3 GFP-EZH2+HA-TRIP12

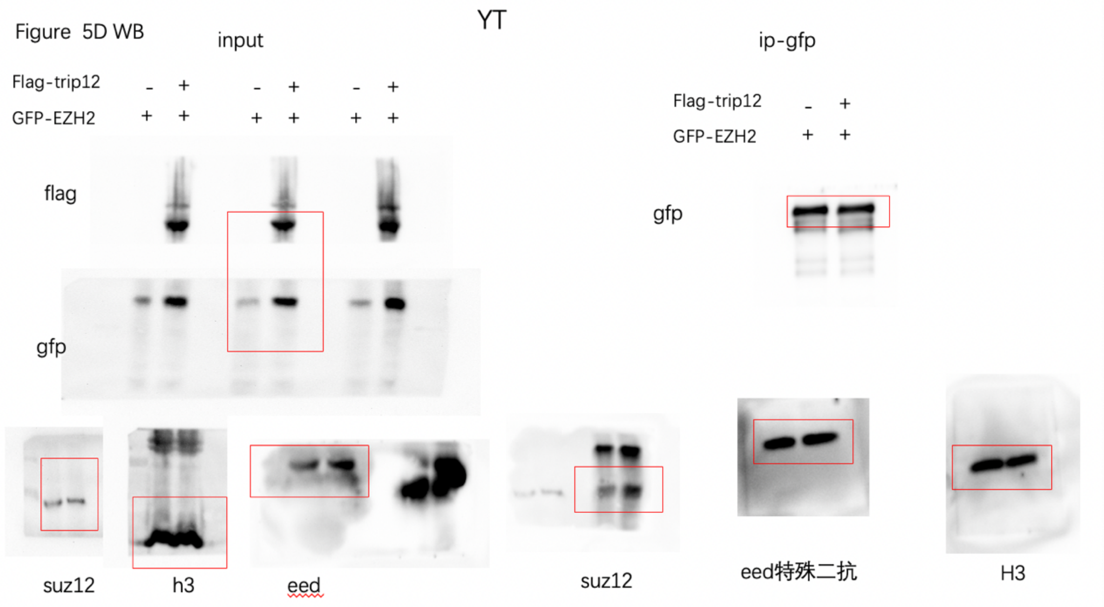
Figure 5D WB
input
YT
ip-gfp
Flag-trip12 - + - + - +
GFP-EZH2 + + + + + +
flag
gfp
Flag-trip12 - +
GFP-EZH2 + +
gfp
suz12
h3
eed
suz12
eed特殊二抗
H3

Figure 5E WB

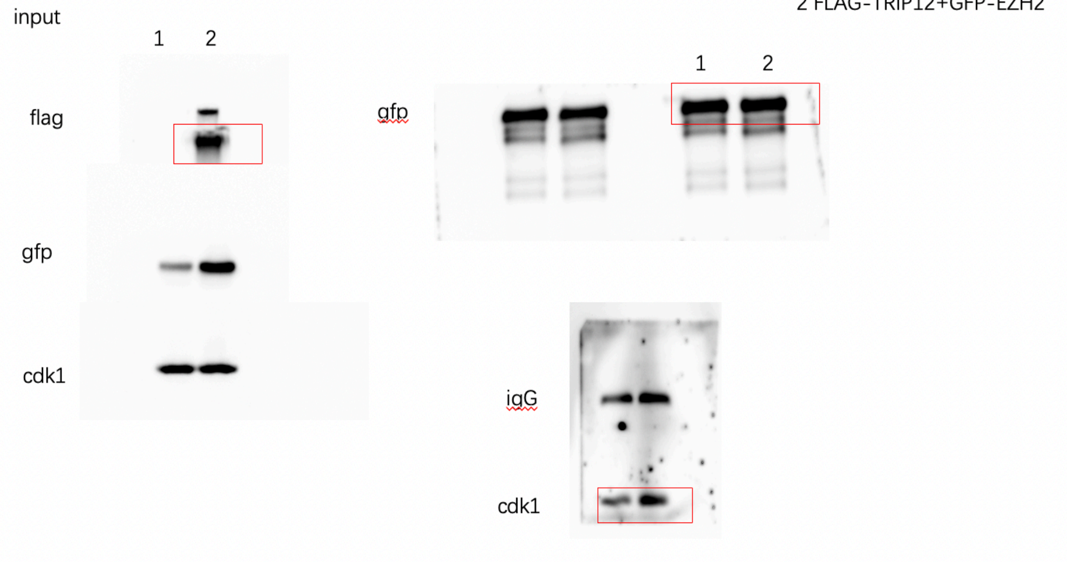

Figure 5F WB

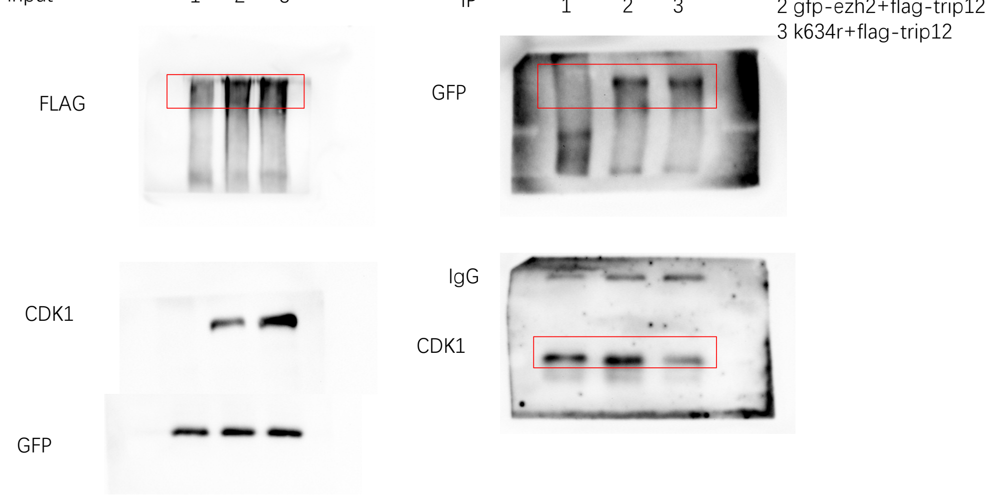

Figure 5G WB

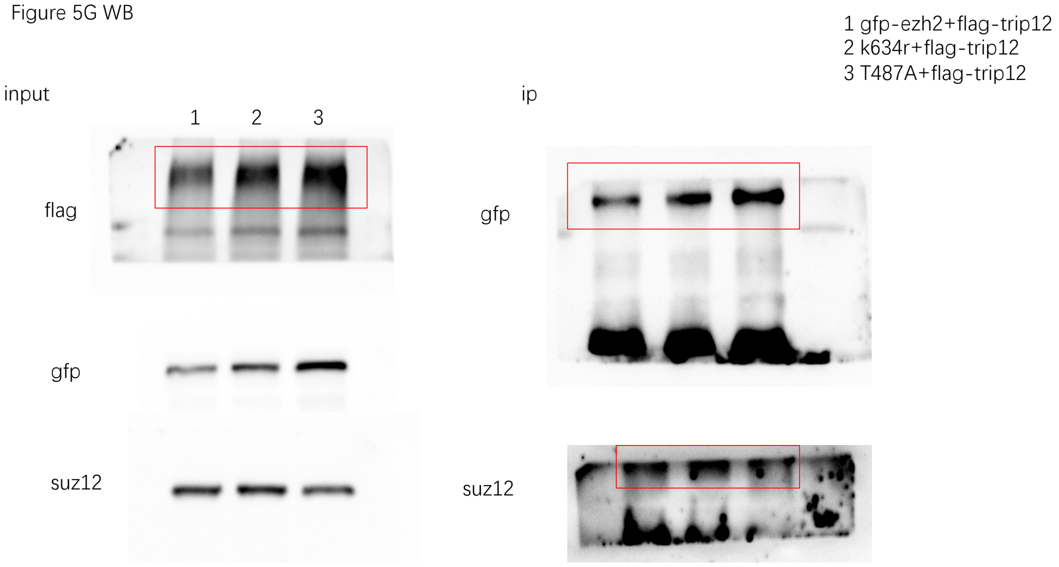

Figure 6A WB
YT

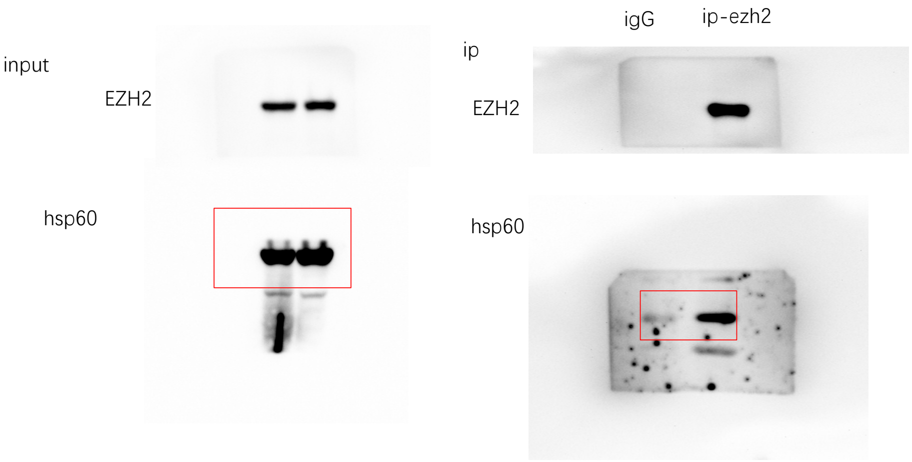

Figure 6A WB

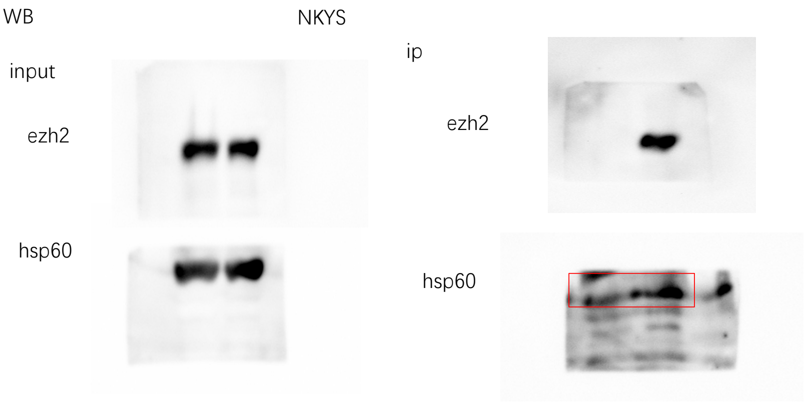

Figure 6B WB

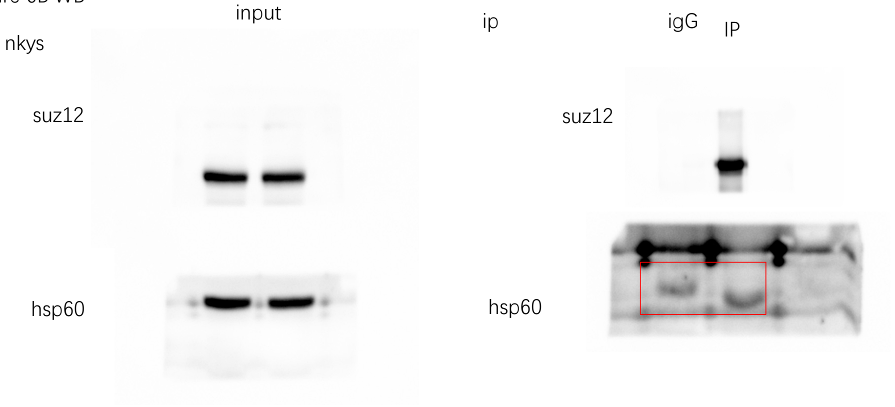

Figure 6B WB

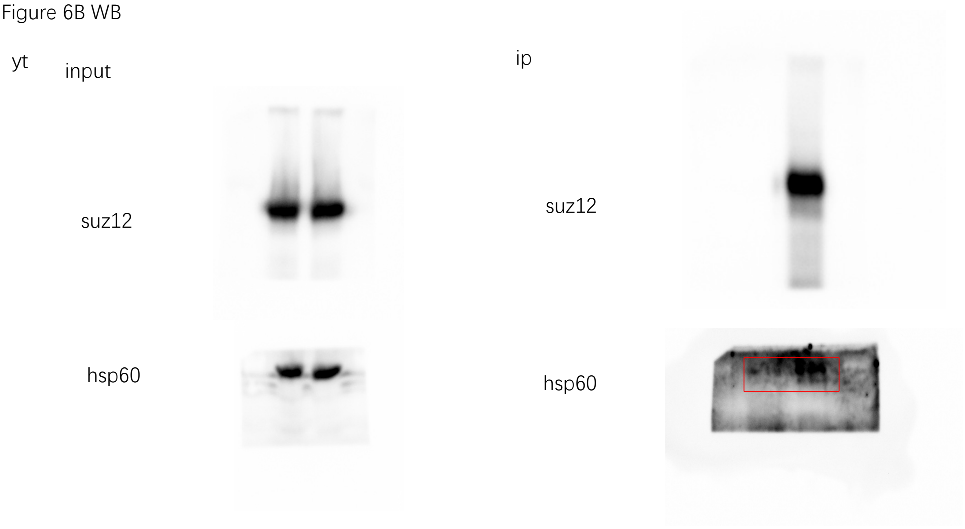

Figure 6C WB

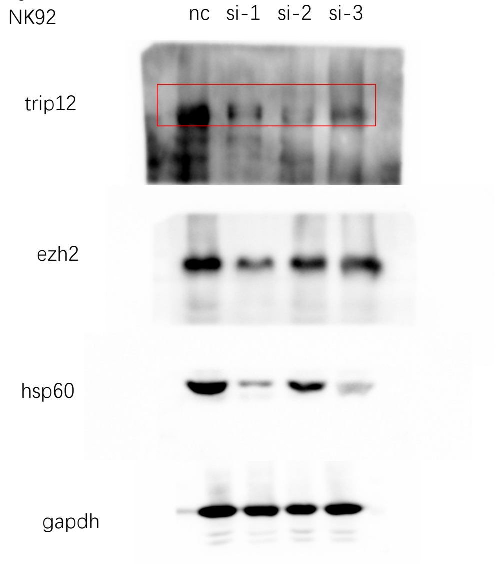

Figure 7A WB

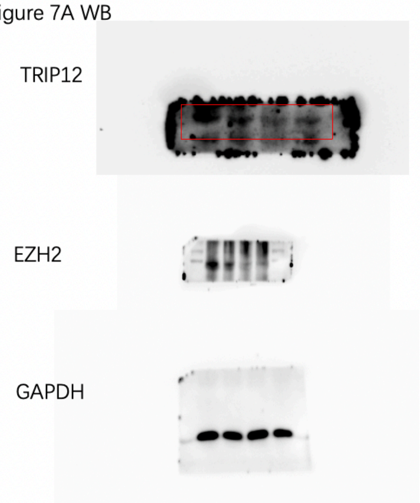

Figure 7B WB

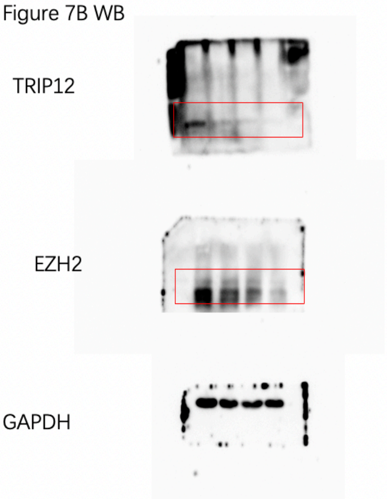

Supplemental figure 2A

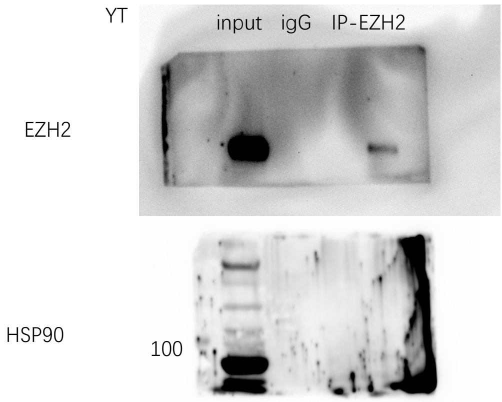

Supplemental figure 2B

YT

input

ezh2

hsp60

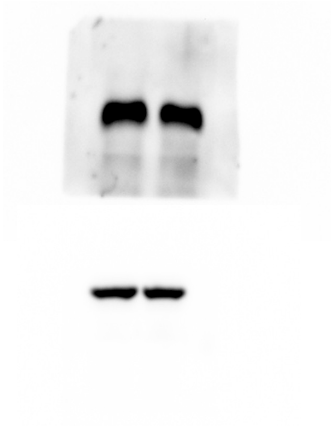

ip

ezh2

hsp60

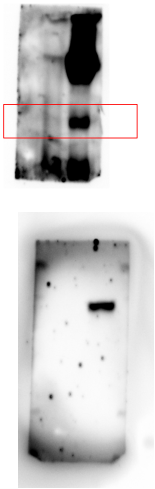

Supplemental figure 2B

nk92

input

hsp60

ip

hsp60

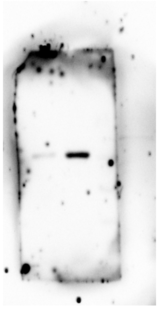

EZH2

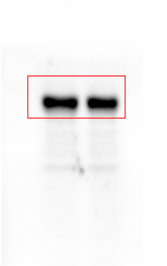

EZH2

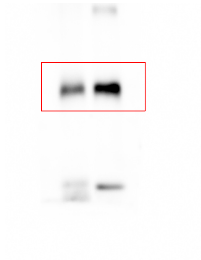

Supplemental figure 2C

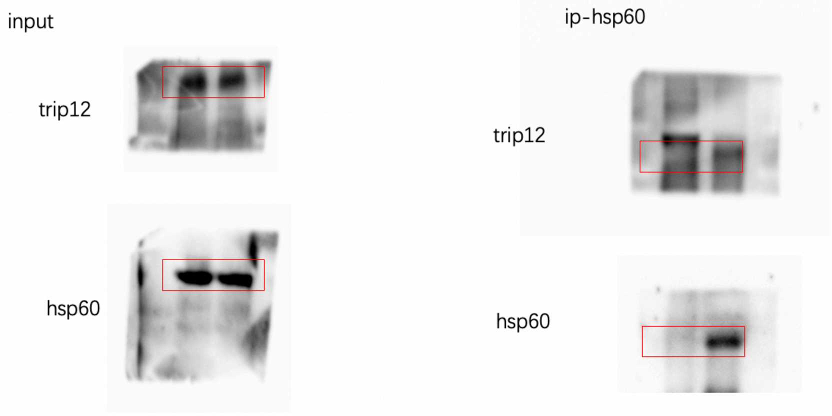

Supplemental figure 2D

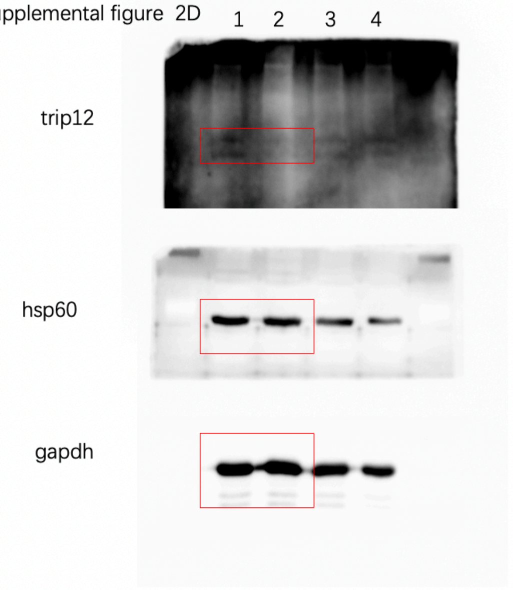

1 yt you nc
2 yt you si-trip12
3 yt wu nc
4 yt wu si-trip12

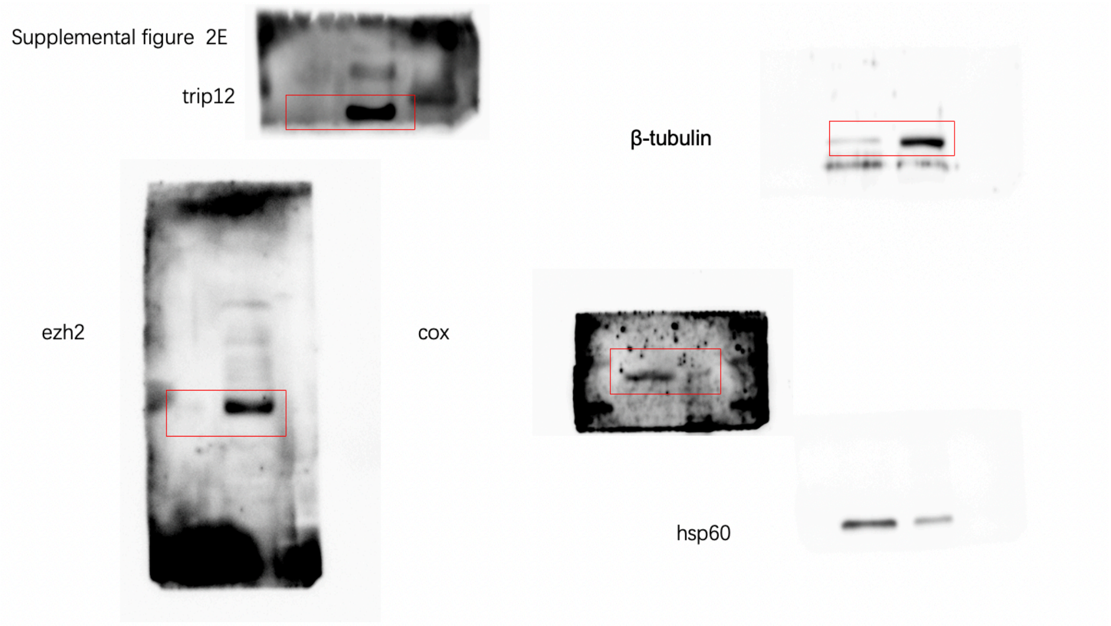
Supplemental figure 2E
trip12
β-tubulin
ezh2
cox
hsp60

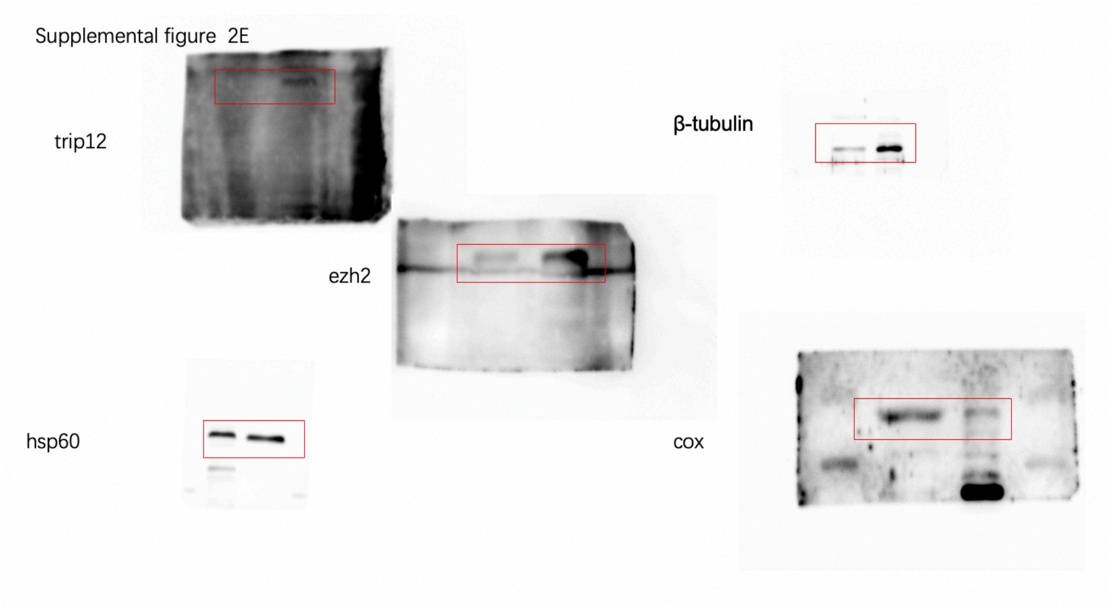
Supplemental figure 2E
trip12
β-tubulin
ezh2
hsp60
cox
